# Multi-Frame Reconstruction via Clutter Filtering for Computational Contrast-Enhanced Ultrasound Microflow Imaging

**DOI:** 10.3390/jimaging12100511

**Published:** 2026-10-14

**Authors:** Dongdong Liang, Daichao Chen, Xiaoyu Qian, Yuantong Zhong, Yu Xia, Jue Zhang

**Affiliations:** 1 The Academy for Advanced Interdisciplinary Studies, Peking University, Beijing 100091, China; ldd2021@stu.pku.edu.cn (D.L.); chendaichao@pku.edu.cn (D.C.); xiaoyu.qian@pku.edu.cn (X.Q.); zhongyuantong@stu.pku.edu.cn (Y.Z.); 2 School of Mechanics & Engineering Science, Peking University, Beijing 100091, China; rain@stu.pku.edu.cn

**Keywords:** blood flow imaging, beamforming, inverse problem, regularization, flow-related signals

## Abstract

Objective: Existing computational reconstruction methods for plane-wave ultrasound imaging (PWI) often neglect the interference of strong tissue signals with weak flow-related scattering signals in microflow imaging. This study aims to develop a multi-frame computational ultrasound reconstruction technique that integrates multi-frame sparse constraints with RF signal preprocessing strategies to facilitate the suppression of tissue signals and background noise. Methods: We propose multi-frame reconstruction of contrast-enhanced ultrasound microflow imaging. First, to prevent tissue signals from diluting the sparsity of multi-frame data, we apply singular value decomposition directly to the raw radio-frequency data, attenuating tissue-dominated components before beamforming. This yields sparser data and increases the relative contribution of flow-related components in the optimization. Results: The method was evaluated in contrast-enhanced microflow simulations and an exploratory in vivo experiment involving one rabbit. Under the tested conditions, VrUSi reduced background signals and produced narrower apparent intensity profiles than delay-and-sum and single-frame computational reconstruction. In the single-rabbit experiment, VrUSi yielded higher image-domain BCR values and smaller apparent-profile FWHM values across the analyzed temporal windows. These metrics are interpreted as background suppression and apparent profile compactness under the tested conditions, rather than as evidence of improved physical contrast or spatial resolution. The findings support feasibility only for visualizing flow-related structures in the tested rabbit lower-limb acquisition.

## 1. Introduction

Plane-wave ultrasound imaging (PWI) has emerged as a powerful imaging modality for real-time visualization of microflow dynamics due to its ultrahigh frame rate capability [1,2,3]. However, achieving high-quality images from PWI requires accurate reconstruction algorithms capable of exploiting the rich information contained in raw radiofrequency (RF) data [4,5].

The delay-and-sum (DAS) algorithm remains the most widely used beamforming method in PWI because of its simplicity and computational efficiency [6,7]. Nevertheless, DAS relies on simplified assumptions, including homogeneous sound velocity and ideal noise-free propagation conditions. In practical imaging scenarios, acoustic aberrations caused by heterogeneous tissues, as well as electronic and physiological noise, introduce deviations between measured RF signals and actual scatterer distributions [8,9]. Consequently, DAS suffers from degraded contrast and resolution, particularly in challenging environments involving weak blood flow signals.

Recently, computational reconstruction methods have provided an alternative framework by formulating ultrasound beamforming as an inverse problem [10]. Instead of directly applying geometric delay corrections, these methods construct a forward imaging model describing the relationship between tissue reflectivity and measured RF data and recover the underlying image through optimization algorithms [11,12]. By incorporating point spread function (PSF) information into the forward model and introducing appropriate regularization constraints, these approaches can suppress side-lobe artifacts and improve image quality beyond conventional DAS reconstruction [13,14].

However, existing computational reconstruction approaches mainly focus on recovering individual B-mode frame. This frame-by-frame strategy ignores information among multiple frames in a high-frame-rate PWI sequence. Contrast-enhanced blood-flow data contain sparse signals from microbubbles (MBs) whose distributions vary across frames, so a joint inverse problem can use this information more efficiently than independent framewise reconstructions.

In this study, we propose an RF-SVD-assisted multi-frame (video) computational reconstruction framework, termed VrUSi, for contrast-enhanced microflow imaging in PWI. Direct reconstruction of unfiltered PWI data is challenging because tissue signals dominate the data and reduce the relative sparsity of blood-flow-related information. We therefore employ singular value decomposition (SVD)-based filtering in the RF domain to attenuate tissue clutter before reconstruction. The filtered frames are reconstructed jointly with sparse penalties on the complete image stack.

The contributions of this study are summarized as follows. We formulate contrast-enhanced PWI as a joint multi-frame sparse inverse problem. We introduce SVD filtering before reconstruction to attenuate tissue clutter and enrich flow-related components, thereby changing the sparsity representation and observed convergence behavior. Attenuating tissue-dominated components before solving the inverse problem increases the relative contribution of retained flow-related components to the optimization. The framework combines a physics-based pulse—echo model with multi-frame sparse estimation. We evaluated the proposed method under several imaging conditions, including simulations, clutter-dominated phantom experiments, sparse microbubble trajectory imaging, and an exploratory in vivo experiment in one rabbit. The comparisons are reported as image-domain measurements under the tested conditions rather than as evidence of general superiority. In the single-rabbit experiment, VrUSi yielded higher BCR and smaller apparent-profile FWHM values; these findings indicate background suppression and apparent profile compactness, respectively.

## 2. Related Works

### 2.1. Computational Reconstruction Methods for Ultrasound Imaging

Traditional DAS beamforming reconstructs ultrasound images by applying propagation delays calculated under assumed acoustic conditions. Although computationally efficient, DAS does not explicitly model the ultrasound imaging process and therefore cannot compensate for acoustic aberrations or complex noise distributions.

To overcome these limitations, inverse-problem-based reconstruction methods have been developed. Kruizinga et al. and Brown et al. introduced computational ultrasound imaging (cUSi), where ultrasound reconstruction was reformulated as an optimization problem based on a forward convolution model [13,14]. By incorporating PSF information into the system matrix, these methods improved spatial resolution and reduced side-lobe artifacts compared with DAS.

Berthon et al. further derived an analytical solution for inverse reconstruction and demonstrated that matrix-based reconstruction could achieve real-time processing while improving image quality under coded acoustic excitation [15]. However, these approaches rely strongly on accurate forward models, and their performance can deteriorate when the assumed PSF or acoustic parameters deviate from experimental conditions.

Regularization strategies play a critical role in stabilizing inverse reconstruction. Ozkan et al. systematically investigated different regularization constraints, including spatial envelope sparsity, spatial smoothness, spectral sparsity, and spectral smoothness, demonstrating that prior assumptions regarding image characteristics can influence reconstruction performance [11]. More recently, Goudarzi et al. introduced data-driven denoising regularization, replacing predefined mathematical sparsity assumptions with learned noise-image orthogonality constraints [16]. Although these studies improved single-frame reconstruction, they primarily exploit spatial or spectral priors and do not utilize multi-frame information available in ultrafast ultrasound sequences.

### 2.2. Exploiting Multi-Frame Information in ULM Imaging

In contrast to conventional beamforming, ultrasound localization microscopy (ULM) has demonstrated the significant value of multi-frame information by exploiting MB trajectories across multiple frames. Yin et al. and Zhang et al. introduced trajectory-based PSF optimization methods, where MB motion patterns were incorporated to improve localization accuracy [17,18]. Leconte et al. further proposed a tracking-first strategy, using MB trajectories as constraints before localization, thereby improving reconstruction robustness [19].

Beyond trajectory tracking, Yan et al. extended deconvolution methods into three-dimensional spatiotemporal reconstruction and introduced temporal regularization terms to improve MB localization accuracy [20]. These studies collectively demonstrate that multiple frames provide valuable information beyond individual-frame reconstruction.

However, most multi-frame reconstruction strategies have been developed for ULM localization rather than ultrasound beamforming. Their application to inverse beamforming remains largely unexplored.

### 2.3. Integration of Multi-Frame Sparsity into Ultrasound Reconstruction

The SUSHI method proposed by Bar-Zion et al. represents one of the earliest attempts to incorporate multi-frame sparsity into ultrasound reconstruction [21]. By constraining vascular structures accumulated over multiple frames, SUSHI achieved improved super-resolution reconstruction. Nevertheless, SUSHI requires pulse inversion (PI) imaging, resulting in substantially reduced frame rates compared with PWI. Furthermore, because PI effectively suppresses tissue signals during acquisition, its strategy cannot be directly transferred to conventional PWI datasets where tissue clutter dominates RF measurements.

In PWI-based contrast-enhanced ultrasound imaging, tissue signals represent a major challenge for exploiting multi-frame sparsity. Strong clutter components reduce the sparsity of vascular and flow signals, causing optimization algorithms to prioritize reconstruction of high-energy tissue components rather than weak blood-flow structures [22,23]. Therefore, an effective framework is required to isolate blood-flow signals before applying these constraints.

### 2.4. Positioning of the Present Study

Existing studies have demonstrated two complementary directions: physics-based inverse reconstruction improves ultrasound image formation by incorporating forward models and regularization, whereas ULM methods reveal the value of exploiting multi-frame sparsity information. However, these two concepts have rarely been integrated with PWI beamforming.

To bridge this gap, this study introduces VrUSi, which combines inverse reconstruction with multi-frame sparsity. By applying SVD filtering before optimization, tissue clutter is attenuated, and the relative contribution of flow-related components is increased before sparse regularization. This enables multi-frame information to directly contribute to beamforming reconstruction and provides a new framework for microflow imaging using PWI.

## 3. Materials and Methods

To enable researchers to obtain a comprehensive understanding of the proposed method and reproduce the results, this study provides a detailed description of the methodology, covering both the theoretical framework and experimental procedures. The Methods Section is organized into three main parts. The first part describes the construction of the system matrix and the formulation of the inverse problem model, as presented in Section 3.1 and Section 3.2. The second part introduces the tissue filtering procedure for raw RF data and the measurement of the actual transducer impulse response using a hydrophone, as described in Section 3.3 and Section 3.4. The third part details the experimental procedures, including simulations, phantom experiments, and in vivo studies, which are presented in Section 3.5, Section 3.6, and Section 3.7, respectively.

### 3.1. Construction of the Pulse—Echo Matrix

Under the assumptions of a constant sound speed (c = 1540 m/s), a homogeneous medium, and negligible attenuation, the construction process of the pulse—echo matrix for a single-angle plane wave can be described as follows.

The pulse—echo response at a spatial point $s_{k}$ corresponding to element $e_{i}$ is expressed as the time-domain convolution between the transducer element’s sensitivity function and the acoustic field signal at $s_{k}$:

(1) $$r_{single}\left(e_{i},s_{k},t\right)=v\left(e_{i},s_{k}\right)⊗f\left(s_{k},t\right)$$

Here, $f(s_{k},t)$ denotes the acoustic field signal at the spatial point $s_{k}$, while $v(e_{i},s_{k})$ represents the sensitivity of the element $e_{i}$ to that point. ${⊗}_{t}$ denotes convolution.

The sensitivity function $v(e_{i},s_{k})$ is computed as the convolution of the element’s impulse response (EIR) with the spatial impulse response (SIR):

(2) $$v\left(e_{i},s_{k},t\right)=EIR\left(e_{i}\right)⊗SIR\left(s_{k}\right)$$

The SIR can be expressed as:

(3) $$\mathrm{SIR}\left(\vec{s_{k}},t\right)=\int_{S}\frac{\delta \left(t-\frac{\left|\vec{s_{k}}-\vec{e_{i}}\right|}{c}\right)}{2\pi \left|\vec{s_{k}}-\vec{e_{i}}\right|}dS$$

where *S* denotes the surface of the element, c is the speed of sound, and δ is the Dirac delta function. In this study, the EIR was experimentally measured using hydrophones, while the SIR was simulated using the Field II software [24,25].

According to the principle of reciprocity, the sound field at a spatial point $s_{k}$ generated by a single-angle plane wave is equivalent to the superposition of acoustic waves emitted from all elements.

(4) $$f\left(s_{k},t\right)=\sum_{i}^{N_{elements}}ex\left(e_{i},t\right)⊗v\left(e_{i},s_{k},t\right)$$

where ex denotes the excitation signal, and $N_{elements}$ refers to the number of elements. Based on this, the pulse—echo matrix for a single element $e_{i}$ under a single-angle plane wave is defined as:

(5) $$p_{e_{i},\theta }=\left[\begin{array}{ccc} r_{\mathrm{single}\;}\left(e_{i},s_{1},t\right) & \ldots & r_{\mathrm{single}\;}\left(e_{i},s_{N_{\mathrm{lateral}}*N_{\mathrm{axial}}},t\right) \end{array}\right]$$

The pulse—echo matrix for the full array under a single-angle plane wave is given by:

(6) $$P_{\theta }=\left[\begin{array}{c} p_{e_{1},\theta }\\ \vdots \\ p_{e_{N_{elements}},\theta } \end{array}\right]$$

For five-angle plane-wave transmissions, the complete pulse—echo matrix becomes:

(7) $$P=\begin{array}{c} P_{\theta_{1}}\\ \left[\begin{array}{c} \vdots \\ P_{\theta_{5}} \end{array}\right] \end{array}$$

Here, the dimensions of the pulse—echo matrix P are $\left[N_{angle}*N_{elements}*N_{t},N_{lateral}*N_{axial}\right]$. $N_{angle}$ denotes the number of angles used in the multi-angle plane-wave imaging, $N_{t}$ denotes the length of the time-domain RF signal, $N_{lateral}$ indicates the number of pixels along the lateral direction of the image, and $N_{axial}$ corresponds to the number of pixels along the axial direction.

**Figure 1:**
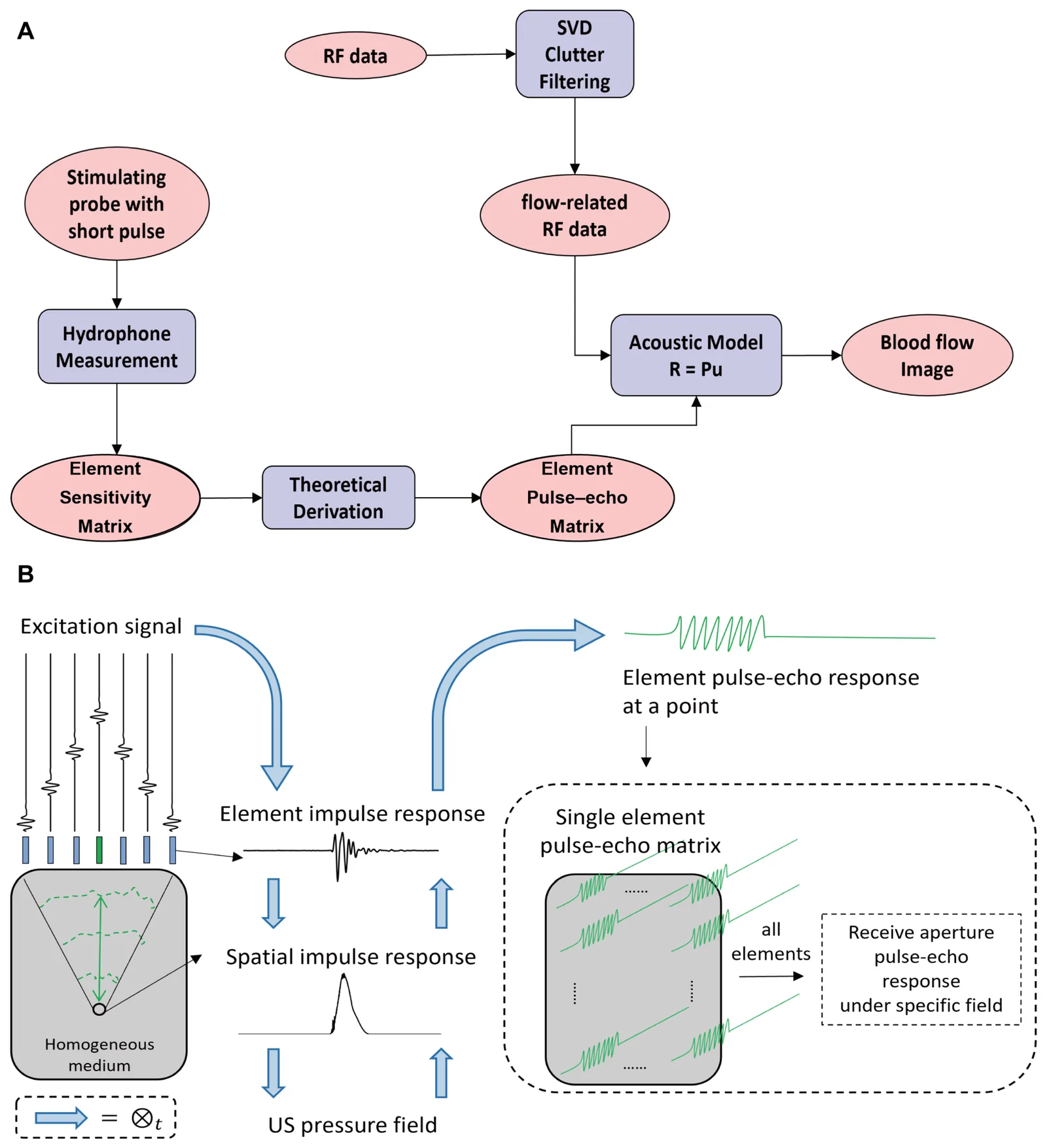
Overview of the proposed acoustic-model-based sparse reconstruction framework. (**A**) Workflow of the proposed method. The element sensitivity matrix was experimentally measured using hydrophone recordings and used to construct the element pulse—echo matrix through theoretical modeling. The acquired RF data were processed by SVD clutter filtering and reconstructed using the acoustic model R = Pu with sparsity constraints to obtain an image of the retained flow-related components. (**B**) Construction of the element pulse—echo matrix. The acoustic response of each element was modeled by combining the element impulse response and spatial impulse response. The pulse—echo responses between all transmit—receive element pairs and imaging locations were calculated to form the system matrix.

Figure 1 illustrates the proposed acoustic-model-based sparse reconstruction framework for ultrasound blood flow imaging. Figure 1A illustrates the overall reconstruction pipeline. The element sensitivity matrix was first experimentally characterized using hydrophone measurements under short-pulse excitation. Based on the measured element sensitivity profiles, theoretical derivation was performed to construct the element pulse—echo matrix, which describes the acoustic response between individual transducer elements and spatial locations. The acquired RF data were processed using SVD-based clutter filtering to attenuate tissue-dominated components and retain flow-related components. The filtered RF data were subsequently incorporated into the acoustic model R = Pu′, where P represents the element pulse—echo matrix, R represents the RF data and u’ denotes multiple frames.

The blood flow image was reconstructed by solving a sparsity-constrained inverse problem. Figure 1B shows the construction of the element pulse—echo matrix. A short excitation pulse was transmitted through individual transducer elements, and the corresponding acoustic pressure field in a homogeneous medium was modeled as the convolution between the EIR and SIR. The simulated pressure field was then used to calculate the pulse—echo response between each transmit—receive element pair and spatial point. By repeating this process across all elements and spatial points, the complete pulse—echo matrix can be constructed. The parameters used in this study are as follows: the number of plane-wave transmission angles $\left(N_{angle}=5\right)$; the number of transmitting and receiving elements $\left(N_{elements}=64\right)$; and the size of the imaging region $\left(N_{lateral}=128,N_{axial}=285\right)$.

### 3.2. Modeling the Forward Problem of Beamforming

The corresponding RF data can be obtained by convolving the pulse—echo matrix with the density distribution of the medium. In this study, we define the model as follows:

(8) $$R=Pu^{\prime }$$

where $u^{\prime }$ represents the multiple density distribution images across the entire imaging area, *P* is the weight matrix, or pulse—echo matrix, and *R* represents the RF data. We reconstruct images by solving the least squares of Equation (9):

(9) $$\hat{u}={\mathrm{argmin}}_{u^{\prime }}{∥R-Pu^{\prime }∥}_{2}^{2}$$

Specifically, we adopt a multi-frame joint reconstruction strategy, where a sequence of continuous frames is simultaneously reconstructed. For a series of $n_{f}$ frames, the reconstructed variable $u^{\prime }$ can be reshaped into a matrix of size $\left[n_{lateral}*n_{axial},n_{f}\right]$. Then, we can treat $u^{\prime }$ as a multi-frame matrix. The matrix $u^{\prime }$ encodes the multiple frames’ sparsity information. In addition, we compute the standard deviation along the temporal (slow-time, $n_{f}$) dimension of $u^{\prime }$, denoted as $u_{std}^{\prime }$, which encapsulates sparsity information. The resulting optimization problem and the corresponding regularization term can be expressed as:

(10) $$\hat{u}={\mathrm{argmin}}_{u^{\prime }}{∥R-Pu^{\prime }∥}_{2}^{2}+\tau \left(\frac{{∥u^{\prime }∥}_{1}}{n_{f}}+{∥u_{std}^{\prime }∥}_{1}\right)$$

(11) $$u_{std}^{\prime }=\mathrm{std}\left(u^{\prime },0,2\right)$$

where τ is regularization penalty parameter. Here, $std(u^{\prime },0,2)$ refers to the standard deviation computed across the rows of $u^{\prime }$ in MATLAB, i.e., along the slow-time dimension, $n_{f}$. Specifically, for the multi-frame image $u^{\prime }$ with dimensions of $\left[n_{\mathrm{lateral}}\times n_{\mathrm{axial}},n_{f}\right]$, the standard deviation is calculated for each pixel along the slow-time dimension over $n_{f}$ frames. The resulting $u_{\mathrm{std}}^{\prime }$ has dimensions of $\left[n_{\mathrm{lateral}}\times n_{\mathrm{axial}},1\right]$. To solve this optimization problem, we employ the Two-step Iterative Shrinkage/Thresholding (TwIST) algorithm proposed by José M. Bioucas-Dias et al. [26].

Before solving the inverse problem, the RF data *R* and the system matrix *P* were normalized to the range [0,1]. The inverse problem was then solved using the TwIST algorithm. The reconstruction variable was initialized with a zero matrix having the same dimensions as the unknown $u^{\prime }$. A monotonic decrease in the objective function was enforced, and only updates that did not increase the objective were accepted. The maximum number of iterations was set to 50. Convergence was assessed based on the relative change in the objective function between two consecutive iterations. The optimization was terminated when this change fell below $10^{-2}$. To avoid premature termination, at least five iterations were performed.

Two $L_{1}$-based regularization terms were introduced to constrain the standard-deviation response and the frame-wise sparse response, respectively. The first regularization term was applied directly to the full spatiotemporal reconstruction to promote sparsity in each reconstructed frame. The second term was constructed by calculating the standard deviation across all reconstructed frames and imposing an $L_{1}$ sparsity constraint on the resulting standard-deviation image. The magnitude of the second regularization term was substantially smaller than that of the first because it was calculated from the standard deviation across all frames. Therefore, the two terms were not directly summed. To compensate for this scale mismatch, the first regularization term was divided by the total number of frames before constructing the combined regularization term. This scale compensation brought the two regularization terms to comparable numerical ranges. It also prevented either term from dominating the optimization solely because of its larger absolute magnitude, while preserving their respective constraints on cross-frame sparsity and frame-wise sparse responses.

Algorithm 1 summarizes the complete workflow of the proposed VrUSi method, including the specific values of all parameters and the dimensions of the matrices and tensors involved.
**Algorithm 1** VrUSi Reconstruction Pipeline.**Require:** Time-domain RF data D and system matrix P **Ensure:** Reconstructed image sequence I
1:Set $N_{e}=64$, $N_{x}=128$, $N_{z}=285$, T=10002:Set τ=0.02, α=1.9608, β=3.92123:Set $\varepsilon =10^{-2}$, $K_{\min}=5$, and $K_{\max}=50$4:Extract the selected transmission, receive channels, and time window from all *T* RF frames5:Form the multi-frame observation matrix $Y=[y_{1},\ldots ,y_{T}]$6:Arrange the time-domain responses of all pixels into P7:Compute the effective penalty parameter: $$\tau_{\mathrm{eff}}=\tau \max\left|P^{T}Y\right|$$8:Define the joint objective function: $$F\left(X\right)={∥Y-PX∥}_{2}^{2}+\tau_{\mathrm{eff}}\left({∥\mathrm{std}(X,2)∥}_{1}+\frac{1}{T}{∥X∥}_{1}\right)$$9:Initialize  $X^{\left(-1\right)}=X^{\left(0\right)}=0_{N_{x}N_{z}\times T}$, $L_{0}=1$, and k=010:**repeat**11:    Compute the residual and gradient: $$E^{\left(k\right)}=Y-PX^{\left(k\right)},\;G^{\left(k\right)}=P^{T}E^{\left(k\right)}$$12:    Apply soft-thresholding: $$Z^{\left(k\right)}=S_{\tau_{\mathrm{eff}}/L_{k}}\left(X^{\left(k\right)}+\frac{G^{\left(k\right)}}{L_{k}}\right)$$13:    Update all *T* frames simultaneously: $$X^{\left(k+1\right)}=\left(\alpha -\beta \right)X^{\left(k\right)}+\left(1-\alpha \right)X^{\left(k-1\right)}+\beta Z^{\left(k\right)}$$14:    Apply the monotone backtracking rule to update $X^{\left(k+1\right)}$ and *L_k_*15:    Compute the relative objective change: $$r_{k}=\frac{\left|F\left(X^{\left(k+1\right)}\right)-F\left(X^{\left(k\right)}\right)\right|}{\max\;\left(F\left(X^{\left(k\right)}\right),10^{-12}\right)}$$16:    k←k+117: **until** $\left(k\geq K_{\min}\;\mathrm{and}\;r_{k}\leq \varepsilon \right)$ **or** $k=K_{\max}$18: Reshape all reconstructed frames simultaneously: $$I=\mathrm{reshape}\left(|X^{\left(k\right)}|,N_{z},N_{x},T\right)$$19: **return** I

### 3.3. Measurement of Element Impulse Response

In this study, a 256 channel Verasonics ultrasound platform (Verasonics Inc., Kirkland, WA, USA) was employed in conjunction with a 64-element linear-array probe operating at 8 MHz (element pitch = 0.2 mm, elevation focus = 20 mm, and −6 dB bandwidth of approximately 50%) to evaluate the proposed algorithm under the reported experimental conditions. The setup was used across simulation, phantom, and in vivo experiments. The imaging transmission strategy involved five-angle plane-wave compounding with steering angles of $\left[-\frac{\pi }{12},\frac{\pi }{12}\right]$, a duty cycle (DC) of 0.67, and a pulse comprising 2 cycles. The resulting frame rate was 500 Hz. This transmission protocol was consistently applied in all simulation, phantom, and in vivo experiments.

To construct a realistic pulse—echo matrix, the actual impulse response of the element was measured using a 0.2 mm needle hydrophone (Precision Acoustics Ltd., Dorchester, UK), enabling accurate modeling of the system response function. The procedure for measuring the element impulse response was as follows: 1. The probe was fixed to a mechanical arm that can move in three directions and rotate. The probe was submerged in a water tank, with its surface parallel to the water surface. 2. Initially, the needle hydrophone was roughly positioned beneath the probe by visual observation, with the target position set at (X = 0, Y = 0, Z = 10 mm). 3. Based on the plane-wave imaging results from the probe, the needle hydrophone position was further adjusted with precision. 4. A focused pulse with the following parameters was used to excite all elements of the probe: DC = 0.3, cycles = 0.5. The focus position is (X = 0, Y = 0, Z = 10 mm). 5. The needle hydrophone collected the signals, which were recorded by an oscilloscope with a sampling rate of 4 GHz. Each collected signal was averaged 32 times to reduce noise. The recorded signal was treated as the element impulse response for all elements of the probe.

**Figure 2:**
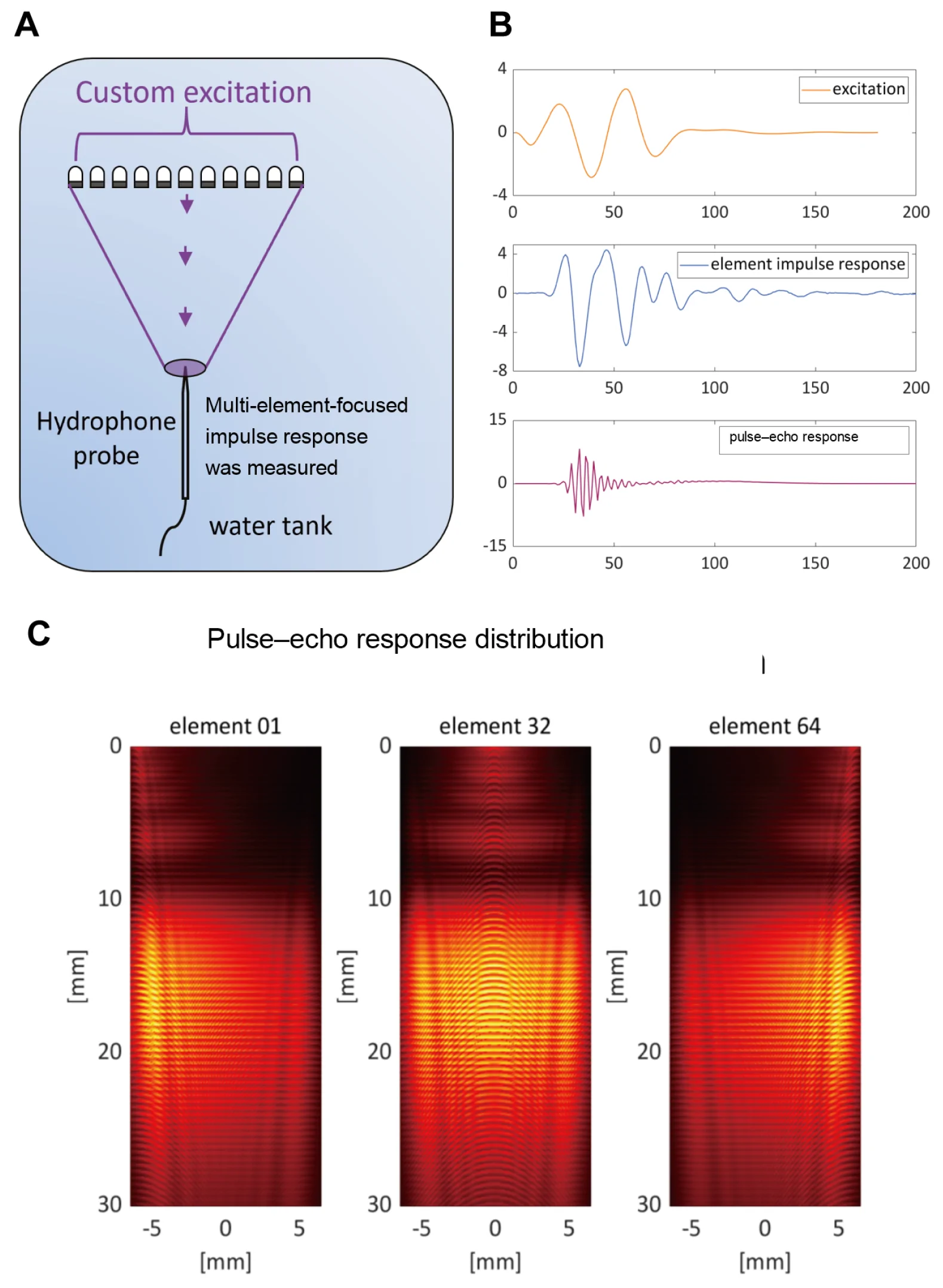
The process of measuring EIR signal. (**A**) Setup for hydrophone measurements. (**B**) Representation of the element excitation signal (yellow), EIR signal (blue), and element pulse-echo response signal (red). (**C**) Spatial distribution of the pulse-echo response for the 1th, 32th, and 64th elements.

The measurement process of the EIR signal is illustrated in Figure 2A. Initially, all elements were excited using a custom-designed signal, shown as the yellow curve in Figure 2B, with parameters: DC = 0.3, 0.5 cycles, and a center frequency of 7.8 MHz. Subsequently, a hydrophone was employed to measure the focused response from all 64 array elements. The corresponding EIR of a single element is depicted by the blue curve in Figure 2B. Based on this measurement, the pulse—echo signals for an individual element were then calculated. The red curve in Figure 2B illustrates the pulse—echo signal of one representative element. Further, the pulse—echo responses from each element to all spatial points were computed to analyze spatial characteristics. Figure 2C presents the spatial intensity distributions of the pulse—echo responses for the 1th, 32th, and 64th elements under a $0^{\circ }$ plane-wave sound field. It can be observed that each element exhibits a stronger response to the region located directly beneath it, with the response depth aligning with the probe’s elevational focus at 20 mm.

### 3.4. SVD Filtering of Raw RF Data

Previous studies typically applied SVD to in-phase/quadrature (IQ) signals after beamforming to suppress tissue signals, leveraging the spatio-temporal coherence differences between tissue and blood flow [27,28]. In contrast, this study applies the SVD technique directly to raw RF data (RF-SVD) prior to beamforming. Specifically, the acquired three-dimensional (3D) RF dataset, represented as rf $\left[n_{t},n_{e},n_{f}\right]$—corresponding to time, element, and frame dimensions—is reorganized into a two-dimensional (2D) Casorati matrix. This is achieved through dimensional reconstruction, forming a spatiotemporal matrix *R*$\left[n_{t}*n_{e},n_{f}\right]$. Such matrix construction has been effectively employed in other imaging modalities, providing a proven framework for spatiotemporal analysis [29,30]. SVD is then performed on the reconstructed matrix *R*:

(12) $$R=U∆V^{*}=\sum_{i}^{\mathrm{Rank}(R)}\lambda_{i}U_{i}V_{i}$$

Let *U* and *V* be orthogonal matrices, and let ∗ denote the complex conjugate. Each column of *U* and *V* represents the spatial and temporal singular vectors of the matrix *R*, respectively, while the diagonal elements of λ correspond to the singular values. After decomposition, components associated with large singular values—typically representing highly coherent tissue signals—are selectively removed to retain components with lower spatiotemporal coherence, including flow-related scattering signals.

(13) $$R_{\mathrm{filtered}}=R-\sum_{i}^{n}\lambda_{i}U_{i}V_{i}$$

The retained RF component is denote by $R_{\mathrm{filtered}}$ rather than by $R_{\mathrm{blood}}$ because RF-SVD does not provide strict blood- or microbubble-specific separation.

In this study, SVD filtering is applied to spatio-temporal matrices with $n_{f}=1000$. For the phantom experiment, the top 1% of singular vectors with the highest singular values are removed. In contrast, for the in vivo experiment, the threshold is increased to the top 5% to account for greater tissue complexity.

### 3.5. Simulation Experiment

To compare the contrast-to-noise ratio (CNR) between VrUSi and DAS and to verify the effectiveness of the regularization term in suppressing noise, we conducted numerical simulation experiments. The specific calculation method for CNR is as follows:

(14) $$\mathrm{CNR}=10*\log_{10}\left(\frac{\left|\mu_{\mathrm{in}}-\mu_{\mathrm{out}}\right|}{\sigma_{\mathrm{out}}}\right)\left[\mathrm{dB}\right]$$

$\mu_{in}$ represents the area inside the orange circle, while $\mu_{out}$ represents the area between the orange and blue circles.

Next, we selected multiple scatterers at various depths and plotted their intensity value curves. The full width at half maximum (FWHM) of the scatterers was calculated to quantitatively assess the apparent profile width. For the simulation results, the reported FWHM mean and standard deviation were calculated across the selected scatterers located at multiple depths; the variation reflects depth-dependent differences in their reconstructed apparent profile widths. In this study, DAS beamforming was implemented using the reconstruction function of the Verasonics system. Image post-processing involved adjusting the image to the same dynamic range for display and normalizing the intensity value curve to the range (0, 1). We compared the reconstruction results under three progressively increasing regularization penalty parameters (τ=0,0.002,0.02), collecting 10 frames for each setting. Image post-processing was limited to applying a consistent dynamic range across all reconstructed images to ensure fair comparison.

### 3.6. Phantom Experiment

To demonstrate that the regularization term suppresses both noise and blood flow in the presence of tissue signals—and that the degree of suppression increases with the regularization penalty parameter—we designed a simple and efficient in vitro phantom experiment.

We first fabricated an agarose-based flow phantom with an outer diameter of 3 mm. The fabrication procedure was as follows: a plastic tube with a 3 mm outer diameter was fixed inside a mold, and a mixture of agar powder and deionized water (at a ratio of 1:100) was poured into it. After the phantom solidified, the plastic tube was carefully removed, forming a hollow channel.

To control the flow, a microfluidic pump was employed to deliver an MB solution at a concentration of $1\times 10^{9}\;{\mathrm{mL}}^{-1}$ and a flow rate of $70\;\mathrm{mL}\;h^{-1}$. To mimic tissue signals, a muscle phantom approximately 1 cm thick was placed above the flow phantom. The ultrasound probe was positioned on top of this layer, with acoustic coupling gel applied between the probe and the muscle phantom to ensure effective transmission. For the flow-phantom quantitative results, the BCR was calculated in multiple temporal windows from the same acquisition; the reported mean and standard deviation describe variation across these windows. We employed the blood-to-clutter ratio (BCR) as an image-domain measure of the separation between flow-related signal and clutter in the reconstructed images, rather than as a direct measure of intrinsic physical contrast. The BCR index was calculated as follows:

(15) $$BCR=10*\log_{10}\left(\frac{\left|u_{\mathrm{blood}}-u_{\mathrm{clutter}}\right|}{\sigma_{\mathrm{clutter}}}\right)\left[\mathrm{dB}\right]$$

Here, $u_{\mathrm{blood}}$ and $u_{\mathrm{clutter}}$ denote the mean reconstructed intensities within the predefined blood and clutter ROIs, respectively, and $\sigma_{\mathrm{clutter}}$ denotes the standard deviation within the clutter ROI. No epsilon or truncation value was introduced. When $\sigma_{\mathrm{clutter}}=0$, the BCR was treated as undefined, was not assigned a finite value, and was omitted from quantitative comparison.

We designed a sparse-MB trajectory imaging experiment. A small quantity of MBs was added to deionized water, where they were allowed to drift slowly. Their motion trajectories were then captured over time. We compared the reconstruction results under four progressively increasing regularization penalty parameters (τ=0,0.002,0.02,0.2), collecting 1000 frames for each setting. Image post-processing was limited to applying a consistent dynamic range across all reconstructed images to ensure fair comparison. For each microbubble trajectory, FWHM was evaluated in reconstructions from multiple temporal windows of this single acquisition. The reported mean and standard deviation therefore represent within-acquisition differences among these temporal-window reconstructions, rather than differences among independent acquisitions.

### 3.7. In-Vivo Experiment

The experimental animal was a Japanese big-eared white rabbit (female, 7 weeks old, 1 in total). The rabbit was procured from Charles River Laboratories in Beijing, China. All animal experiments adhered to the Animals (Scientific Procedures) Act of 1986 and received approval from the local Animal Ethics Committee at Peking University (AAIS-ZhangJ-8). Before the experiments, a 10% chloral hydrate solution was used for general anesthesia, with the rabbit receiving an intramuscular injection of $3\;\mathrm{mL}\;{\mathrm{kg}}^{-1}$, followed by depilation of the left hind limb. Subsequently, MBs were injected into the marginal ear vein at a concentration of $1\times 10^{11}\;{\mathrm{mL}}^{-1}$ with a dose of $0.1\;\mathrm{mL}\;{\mathrm{kg}}^{-1}$. Once the MBs had fully perfused the lower extremities, data collection commenced. The rabbit was allowed to breathe freely, and the ultrasound probe was fixed in place using a mechanical arm. We collected 5000 frames of RF data over 10 s from the lower limb of the rabbit.

The previous cUSi and DAS methods both require the reconstruction of B-mode images using the original RF data, followed by the application of SVD filtering to attenuate tissue-dominated components. In contrast, in this study, our VrUSi method utilizes RF data that has already been filtered by SVD, eliminating the need for further SVD processing of the reconstructed images.

For the acquired 5000-frame ultrasound dataset, the data were first divided chronologically into five consecutive segments, each containing 1000 frames. For each 1000-frame segment, tissue clutter and other strong low-frequency components were attenuated using clutter filtering, while dynamic signals primarily associated with blood flow were retained. The signal power was then accumulated over the 1000 frames at each pixel to characterize the local blood-flow-related echo energy, thereby generating a power Doppler image. The same procedure was applied to all five segments, resulting in five consecutive power Doppler images from the original 5000-frame dataset. For visualization, logarithmic compression and normalization were further applied to enhance the display of blood flow signals.

For the in vivo comparison, the same top 5 percent singular-vector cutoff was used in all four workflows, but the filtering location differed. For DAS and raw-RF cUSi, SVD filtering was applied after B-mode reconstruction; for RF-SVD+cUSi and VrUSi, it was applied to the raw RF data before reconstruction. Subsequently, all four workflows underwent identical post-processing using ImageJ (1.54 version) and Matlab (2020b) software. This post-processing included the Roll Ball algorithm in Fiji to remove background noise, with a window size set to 80 pixels, and normalization to a consistent dynamic range [31].

We treated the five non-overlapping 1000-frame blocks obtained from the same animal acquisition as within-recording repeated measurements. For each block, we calculated the CNR, BCR, and apparent FWHM for all reconstruction methods. Because all measurements were derived from a single animal and a single recording session, we considered these blocks as technical repetitions rather than independent biological replicates.

Therefore, we no longer report effect size estimates or confidence interval analyses, as these metrics may not be appropriate for assessing biological differences from a single-animal acquisition. Instead, we summarized the performance of each method using descriptive statistics, including the mean value and standard deviation across the five temporal windows. These analyses were intended to describe within-recording variation across the five temporal windows under the given acquisition condition, rather than to provide population-level statistical inference or biological effect estimation. All analyses were performed in Python 3.12 using NumPy and SciPy.

## 4. Results

### 4.1. Simulation Results

**Figure 3:**
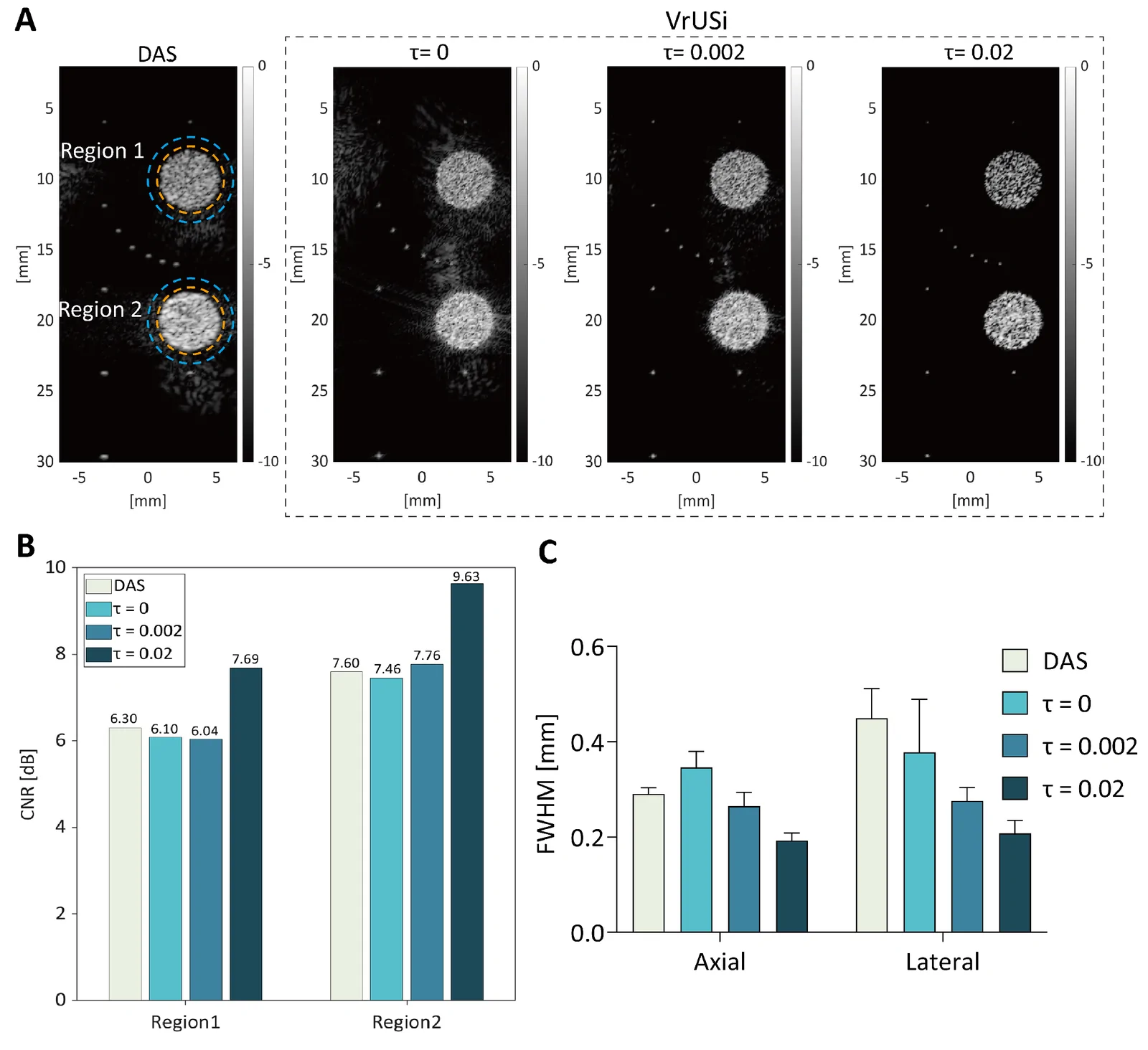
DAS and VrUSi simulation reconstruction results. (**A**) DAS reconstruction results and VrUSi reconstruction results under varying penalty coefficients. (**B,C**) The CNR of DAS and VrUSi images at various depths.

Figure 3A presents the DAS reconstruction results and the VrUSi reconstruction results under varying penalty coefficients for the regularization term. In the absence of regularization (i.e., when τ=0), the VrUSi images exhibited substantial background noise, as shown in Figure 3A. As the penalty coefficient increased, the background noise in VrUSi images progressively decreased. At τ=0.02, low-amplitude background components were strongly suppressed relative to DAS under the matched display settings, as shown in Figure 3A. Figure 3B illustrates the CNR of the VrUSi images at various depths.

As the regularization penalty coefficient increased, the CNR of VrUSi gradually increased. At τ=0.02, VrUSi achieved the highest CNR among all tested configurations. In Region 1, the CNR values were 6.30 dB for DAS, 6.10 dB for VrUSi (τ=0), 6.04 dB for VrUSi (τ=0.002), and 7.69 dB for VrUSi (τ=0.02). In Region 2, the corresponding CNR values were 7.60 dB, 7.46 dB, 7.76 dB, and 9.63 dB, respectively. These results show a higher image-domain CNR with stronger regularization under the evaluated simulation conditions; because regularization directly suppresses low-amplitude components, they do not independently establish an improvement in intrinsic physical contrast.

As shown in Figure 3C, the FWHM decreased with increasing regularization penalty parameter τ. VrUSi achieved the lowest FWHM values at τ=0.02. In the axial direction, the FWHM values were 0.28±0.05 mm for DAS and 0.18±0.05 mm for VrUSi (τ=0.02). In the lateral direction, the corresponding values were 0.46±0.05 mm for DAS and 0.21±0.05 mm for VrUSi (τ=0.02). The reported FWHM mean and standard deviation were calculated across scatterers at multiple depths. The observed variation reflects depth-dependent differences in reconstructed apparent profile width, with deeper scatterers generally exhibiting larger FWHM values. These results indicate that stronger regularization reduced the reconstructed profile width in both axial and lateral directions. It should be noted that sparse regularization can intrinsically narrow reconstructed profiles by suppressing low-amplitude components. Therefore, the reduced FWHM should be interpreted as an improvement in the apparent spatial concentration of reconstructed targets rather than a direct equivalent improvement in the physical resolution limit of the imaging system.

### 4.2. Muscle Flow Phantom Imaging Experiment

**Figure 4:**
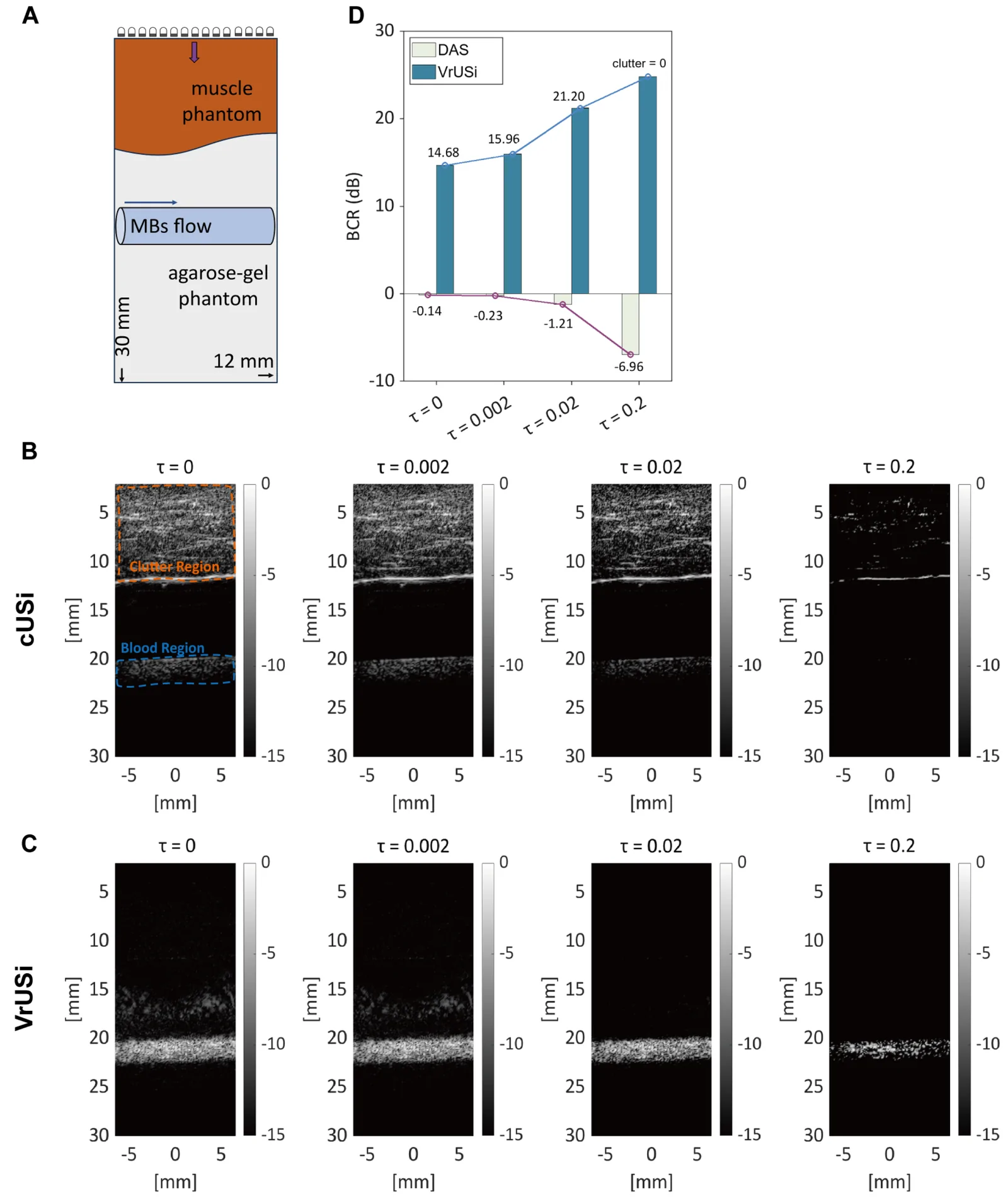
Muscle flow phantom imaging results of cUSi and VrUSi. (**A**) The structural distribution of the phantom. (**B**) Reconstruction results using the cUSi method under varying penalty coefficients. The orange region of interest (ROI) represents the clutter region, while the blue ROI represents the blood region. (**C**) Reconstruction results using the VrUSi method under varying penalty coefficients. (**D**) The trend of BCR index variations for both methods with the penalty coefficients. All images are presented within the same dynamic range.

Figure 4A shows the phantom structure: a 30-mm depth with muscle (0–10 mm) over agarose (10–30 mm) containing 3-mm-diameter channels, perfused with MBs at $1\times 10^{9}\;{\mathrm{mL}}^{-1}$ and $70\;\mathrm{mL}\;h^{-1}$. Figure 4B displays traditional cUSi results: at τ=0 it yields a B-mode image where tissue intensity far exceeds the signals in the MB-filled channel. Increasing τ suppresses both tissue and flow-related components in the channel; at τ=0.02 and τ=0.2, progressively more low-amplitude components are suppressed. The regularization primarily penalizes weak signals without distinguishing tissue, flow-related components, and noise. Figure 4C presents VrUSi results: at τ=0, it produces an image of retained flow-related components with enhanced intensity but some artifacts above the flow region. As τ increases, these artifacts are gradually suppressed, while channel-associated flow-related components remain visible under the tested settings. Figure 4D plots BCR versus τ: cUSi BCR decreases, whereas VrUSi BCR increases. These BCR trends describe image-domain background suppression under the selected settings and do not establish general image-quality superiority or microbubble-specific reconstruction. For VrUSi (τ=0.2), the clutter-region standard deviation was zero, resulting in a zero denominator in the BCR calculation. The corresponding BCR was therefore undefined and was not included as a finite quantitative result. This was caused by the stronger sparsity constraint, which suppressed a large number of weak signals.

### 4.3. MB Trace Experiment

**Figure 5:**
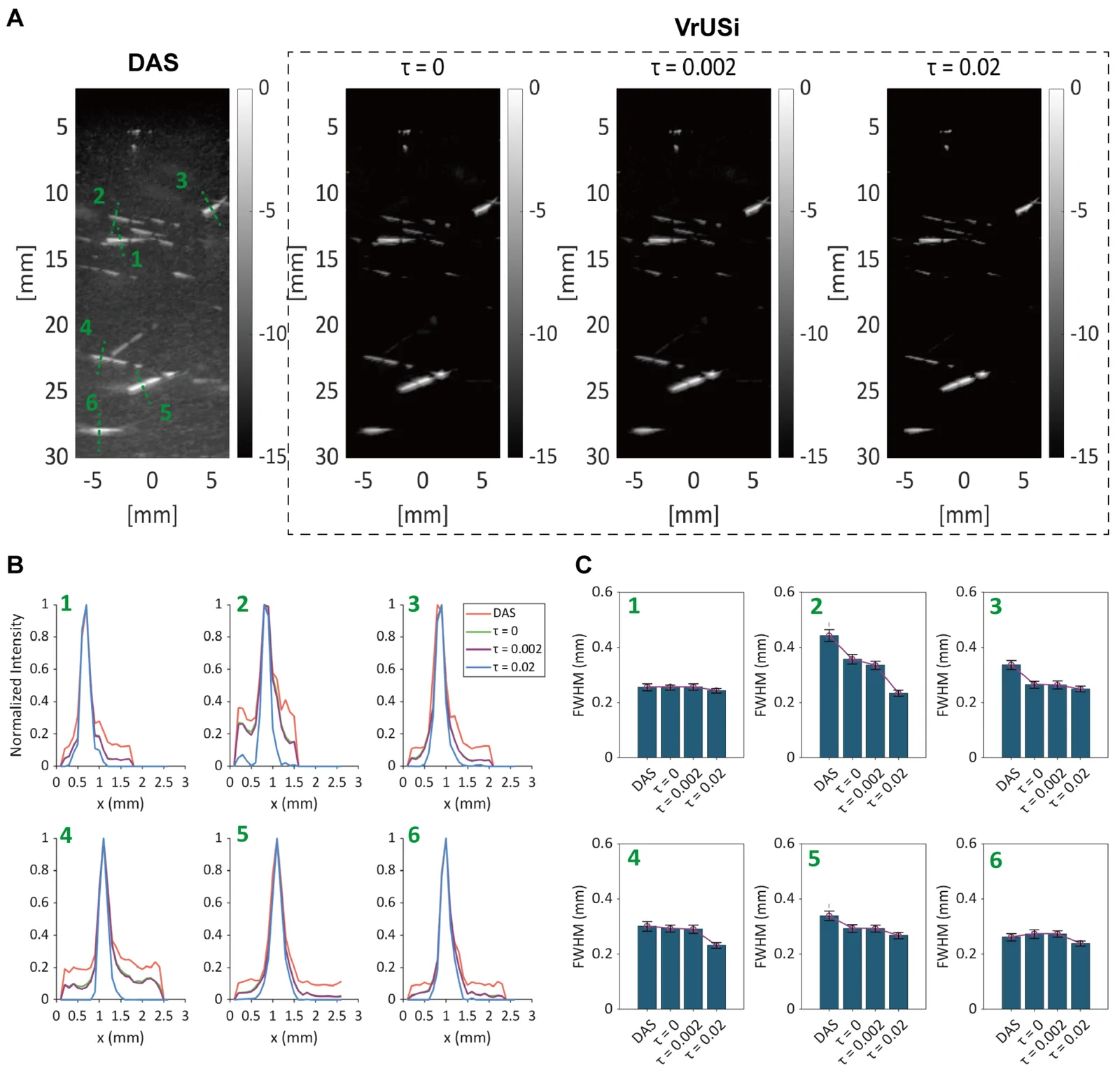
Imaging results from the sparse MB trajectory experiment. (**A**) Reconstructed trajectory like flow related signals using the DAS and VrUSi methods. (**B**) Intensity profiles of selected trajectories. (**C**) Quantitative comparison of FWHM values for both methods.

Figure 5 shows images of trajectory-like flow-related signals in the sparse-MB experiment reconstructed by DAS and VrUSi under varying τ. Figure 5A shows imaging results for six trajectories (green dashed lines), with FWHM measured to assess apparent profile width. Figure 5B presents intensity profiles: as τ increases, trajectory edges sharpen and side fluctuations decrease; VrUSi yields narrower, smoother trajectories than DAS. Figure 5C provides quantitative FWHM comparison: VrUSi FWHM tends to decrease with τ in trajectories 2 and 4, while changing little in trajectories 1, 3, 5, and 6. It should be noted that sparse regularization may narrow the reconstructed profile. Therefore, the reduction in FWHM does not necessarily represent an equivalent improvement in the true physical resolution of the imaging system.

### 4.4. In-Vivo Imaging Experiment

**Figure 6:**
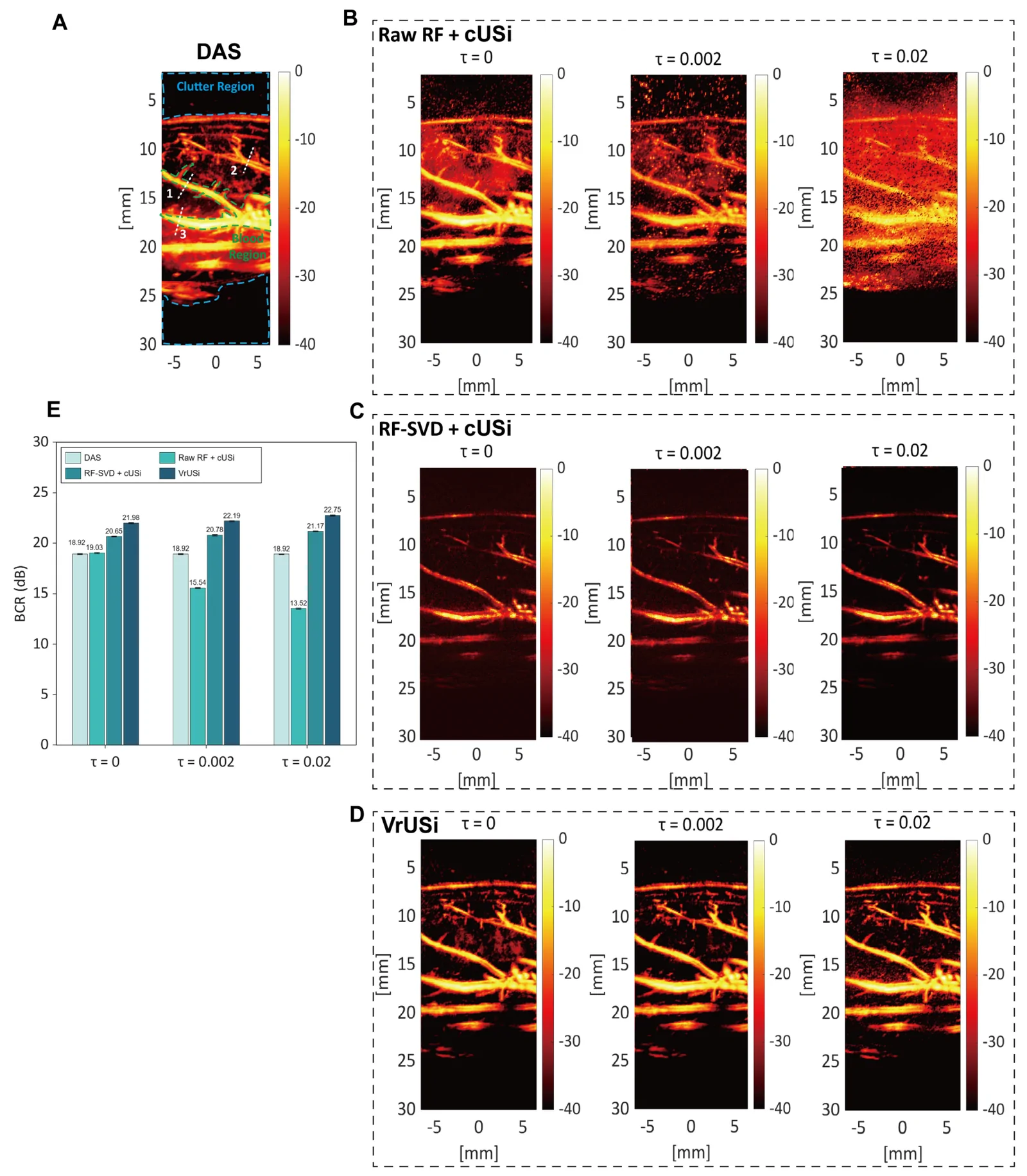
Contrast enhanced blood flow imaging of the rabbit’s lower limb. (**A**) DAS reconstruction results. The tissue dominated component was attenuated, but significant background noise remains in the blood flow area. The green ROI indicates the blood flow region, while the blue ROI corresponds to the clutter region. Three white dashed lines marking blood vessels were employed to evaluate the apparent profile FWHM. (**B**) Contrast enhanced blood flow imaging results using the traditional cUSi (Raw RF data) method with varying regularization penalty parameters. (**C**) Contrast enhanced blood flow imaging results using the cUSi method (RF SVD data) with varying regularization penalty parameters. (**D**) Contrast enhanced blood flow results from the proposed VrUSi method under different regularization penalty parameters. (**E**) Comparison of the BCR indices across the four reconstruction methods.

Phantom experiments showed that most flow-related components in the MB-filled channel were suppressed at τ=0.2. We reconstructed power Doppler images at τ = 0, 0.002, and 0.02 to evaluate the penalty coefficient’s impact on the image-domain signal distribution. Figure 6A shows the DAS power Doppler image. Although DAS effectively suppresses noise in clutter regions, noticeable background noise remains in the blood flow area. Figure 6B shows the conventional cUSi power Doppler image. At τ=0, noticeable background noise is present. As τ increases, background noise becomes more pronounced and BCR decreases. At the higher tested values of τ, cUSi produced higher background levels and a lower BCR than at τ=0 and than DAS under the matched processing conditions. This change occurs because cUSi suppresses both blood flow signals and noise during B-mode reconstruction. After SVD filtering, these suppressed components are retained and amplified, increasing background noise in the final Doppler image. These observations describe the tested image-domain outputs and are not interpreted as evidence of general inferiority. Figure 6C shows cUSi reconstruction performed after RF-SVD. Applying tissue filtering before cUSi increased the image-domain BCR, primarily through stronger background suppression. As τ increased, background noise was further suppressed, resulting in higher BCR values. Figure 6D presents the proposed VrUSi power Doppler image, which shows suppressed background noise while preserving visible vessel-associated structures. Across the tested values of τ, vessel-associated signals remained visible while the background level changed. Figure 6E compares BCR values. The BCR of cUSi (Raw RF data) decreases noticeably with increasing τ and remains lower than that of DAS. VrUSi yielded higher BCR values than cUSi and DAS at the tested parameter values, with relatively small numerical variation across these settings. This within-dataset observation is not evidence of robustness across independent acquisitions.

**Figure 7:**
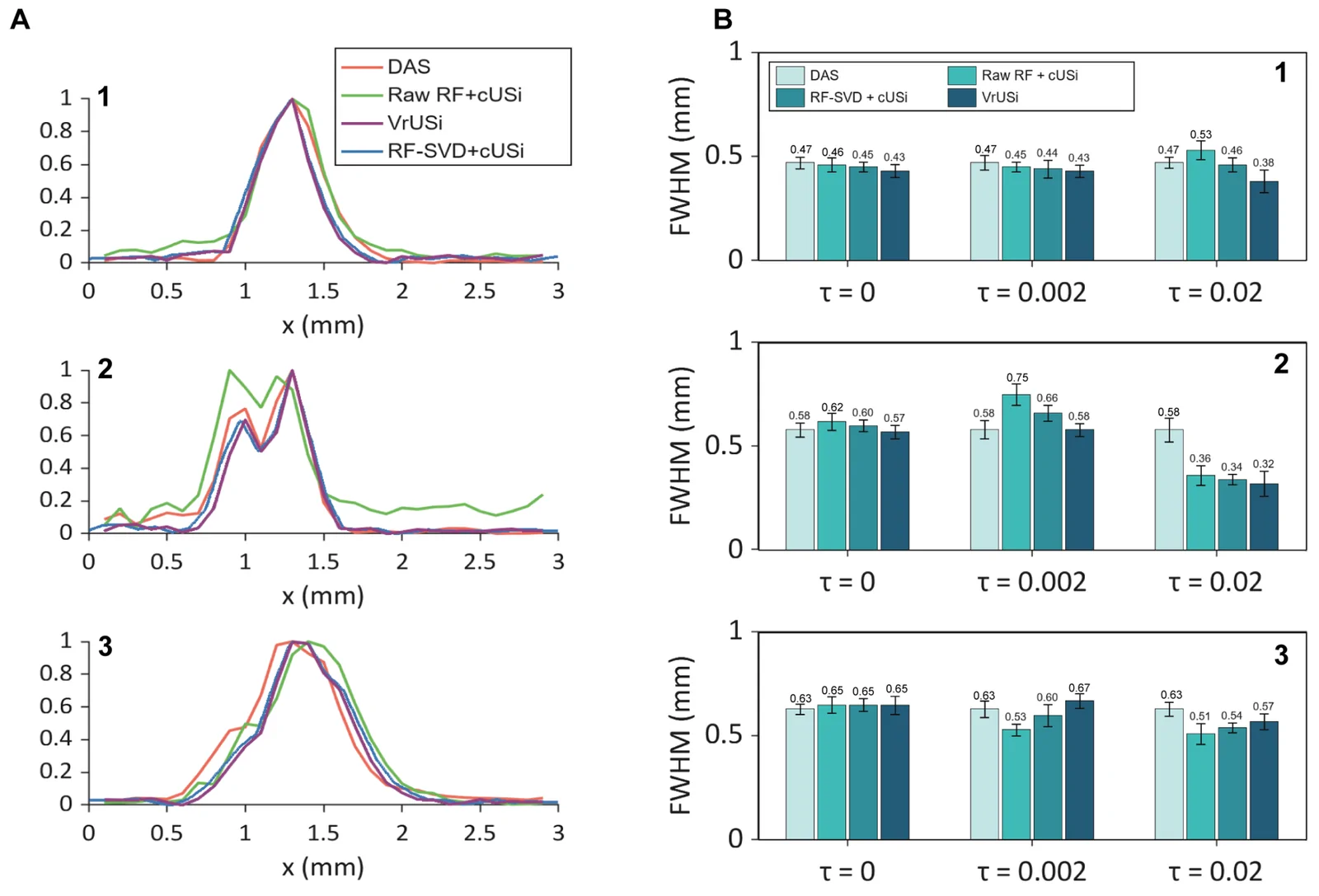
Vessel Intensity Curves and FWHM Results. (**A**) Intensity value curves of different blood vessels reconstructed using four workflows. (**B**) Quantitative comparison of apparent-profile FWHM across the four workflows.

Figure 7A displays intensity curves of three lower limb vessels, comparing VrUSi (τ = 0.02), cUSi (Raw RF data) (τ = 0), cUSi (RF-SVD data) (τ = 0) and DAS. For the selected vessel profiles, VrUSi yielded smaller apparent profile widths than DAS and cUSi, while the overall curve shapes were similar. Figure 7B shows FWHM results. cUSi (Raw RF data) FWHM values exhibited different trends with varying τ: the FWHM of the first vessel increased while that of the third vessel decreased. This discrepancy is mainly due to background noise affecting FWHM estimation for cUSi.

In contrast, VrUSi achieved lower apparent FWHM values compared with DAS under the evaluated conditions. As the regularization parameter increased, the FWHM of VrUSi gradually decreased, with the lowest values obtained at τ = 0.02. For the three vessels, the FWHM values of DAS were 0.47 mm, 0.58 mm, and 0.63 mm, respectively. In comparison, VrUSi with τ = 0.02 yielded FWHM values of 0.38 mm, 0.32 mm, and 0.57 mm, respectively. These results indicate that stronger regularization reduced the apparent width of reconstructed vessel profiles. However, sparse regularization can intrinsically suppress low-amplitude components and concentrate reconstructed signals. Therefore, the reduced FWHM should be interpreted as an improvement in apparent profile compactness rather than a direct equivalent improvement in the intrinsic physical resolution of the imaging system.

Table 1 summarizes the quantitative performance of DAS, raw-RF cUSi, RF-SVD+cUSi, and the proposed VrUSi method across simulation, sparse-microbubble imaging, flow-phantom, and in vivo experiments.

In the simulation, VrUSi achieved CNR values of 7.69±0.10 dB and 9.63±0.10 dB in Regions 1 and 2, respectively, exceeding the corresponding DAS values of 6.30±0.10 dB and 7.60±0.10 dB. VrUSi also produced narrower apparent image profiles, with axial and lateral FWHM values of 0.18±0.05 mm and 0.21±0.05 mm, compared with 0.28±0.05 mm and 0.46±0.05 mm for DAS. For these simulation FWHM results, the mean and standard deviation were calculated across scatterers at multiple depths, and the variability reflects depth-dependent differences in reconstructed apparent profile width. In the sparse-microbubble experiment, the FWHM decreased from 0.34±0.10 mm with DAS to 0.24±0.05 mm with VrUSi. For each microbubble trajectory, the FWHM mean and standard deviation were calculated across reconstructions from different temporal windows of the same acquisition; the variability therefore represents within-acquisition temporal-window differences.

For the flow-phantom experiment at τ = 0.02, VrUSi yielded a BCR of 21.20±0.05 dB, whereas raw-RF cUSi produced a BCR of −1.21±0.05 dB, indicating stronger image-domain background suppression under the evaluated conditions rather than an independently established improvement in intrinsic physical contrast. In vivo, VrUSi achieved the smallest apparent-profile FWHM (0.42±0.10 mm) and the highest image-domain BCR (22.75±0.10 dB) among the four methods. By comparison, DAS, raw-RF cUSi, and RF-SVD+cUSi yielded FWHM values of 0.56±0.10, 0.47±0.10, and 0.45±0.10 mm, together with BCR values of 18.92±0.10, 13.52±0.10, and 20.84±0.10 dB, respectively. VrUSi also reduced the computation time to $8.63\times 10^{3}$ s, compared with $1.18\times 10^{5}$ s for raw-RF cUSi and $1.13\times 10^{5}$ s for RF-SVD+cUSi. For both the flow-phantom and in vivo experiments, the reported means and standard deviations quantify variation among temporal windows within their respective single acquisitions. In the in vivo experiment, these windows were the five non-overlapping 1000-frame blocks defined above. These values therefore describe within-acquisition technical variation rather than variation among independent biological replicates.

### 4.5. Ablation Experiments: The Impact of Regularizations on Imaging and Computation

To quantitatively compare the imaging differences among three schemes—using raw RF data, using SVD-filtered RF data, and the proposed VrUSi method (i.e., SVD filtering followed by spatiotemporal constraint imposition)—we designed ablation experiments related to regularization and evaluated the reconstruction results under different conditions.

**Figure 8:**
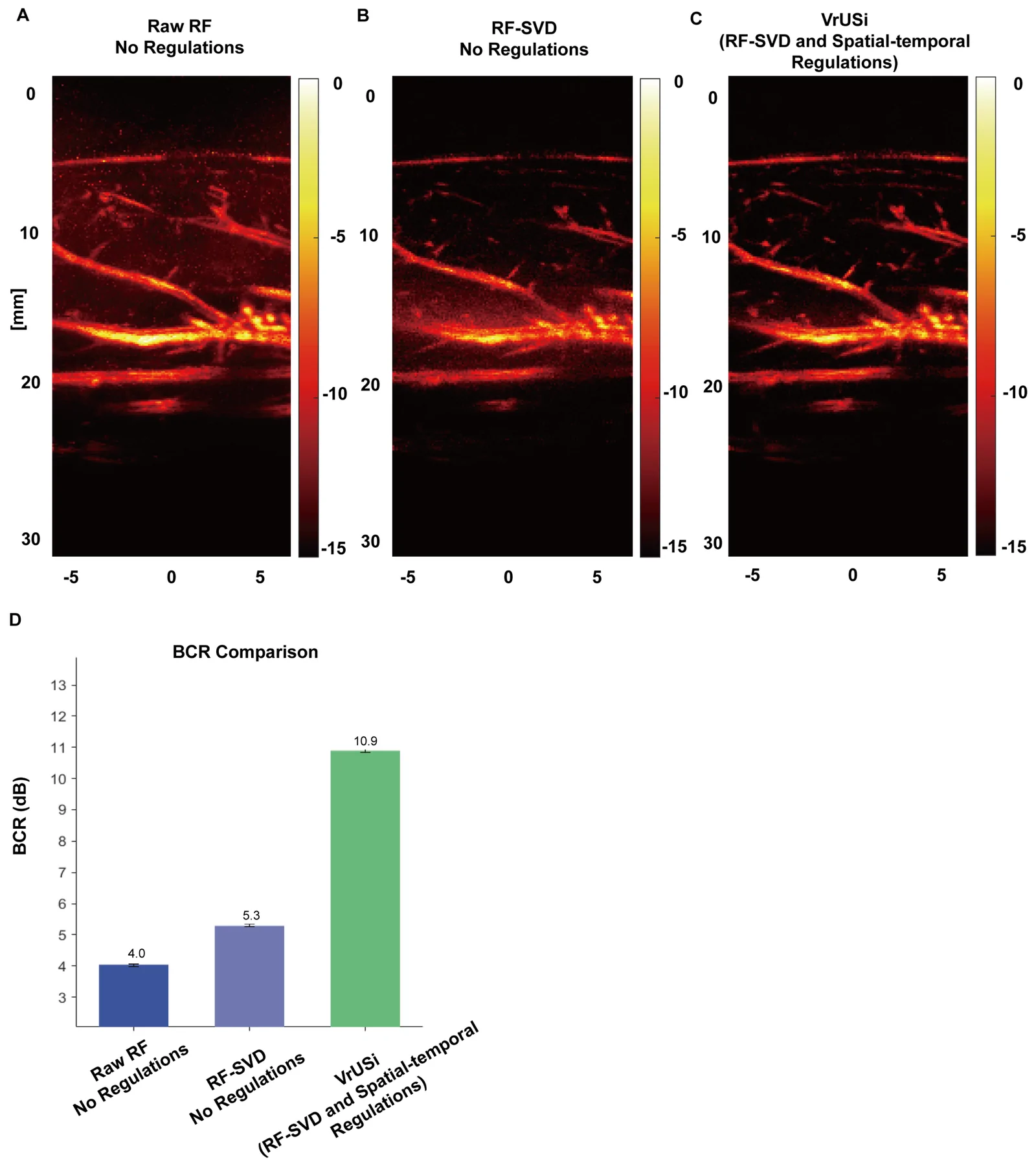
VrUSi imaging results under different regularization settings. (**A**) Raw RF data without regularization. (**B**) RF SVD filtered data without regularization. (**C**) VrUSi using RF SVD filtered data and spatiotemporal regularization. (**D**) Comparison of BCR values.

Figure 8A shows the reconstruction result using raw RF data without regularization. Blood flow was roughly reconstructed, but background noise was severe and vessel edges were blurred. Figure 8B shows the result using SVD-filtered RF data without regularization. It can be seen that after SVD filtering, the background noise is markedly reduced. This indicates that the RF SVD operation affects the reconstruction. Figure 8C shows the reconstruction result of the proposed VrUSi method. The image has a lower background level, and vessel-associated structures are more visible under the matched display settings. This image-domain comparison demonstrates the effect of adding RF-SVD and regularization under the tested settings; it does not establish proximity to a global optimum. Figure 8D compares the quantitative BCR results for the three cases. Compared with reconstructions using raw RF data and RF-SVD data without regularization, VrUSi achieved higher BCR values under the evaluated conditions. The BCR values were 4.0±0.1 dB for raw RF reconstruction without regularization, 5.3±0.1 dB for RF-SVD reconstruction without regularization, and 10.9±0.1 dB for VrUSi with RF-SVD and regularizations. These results indicate stronger image-domain background suppression with the combination of RF-SVD preprocessing and regularization in this experiment; they do not independently establish an improvement in intrinsic physical contrast.

**Figure 9:**
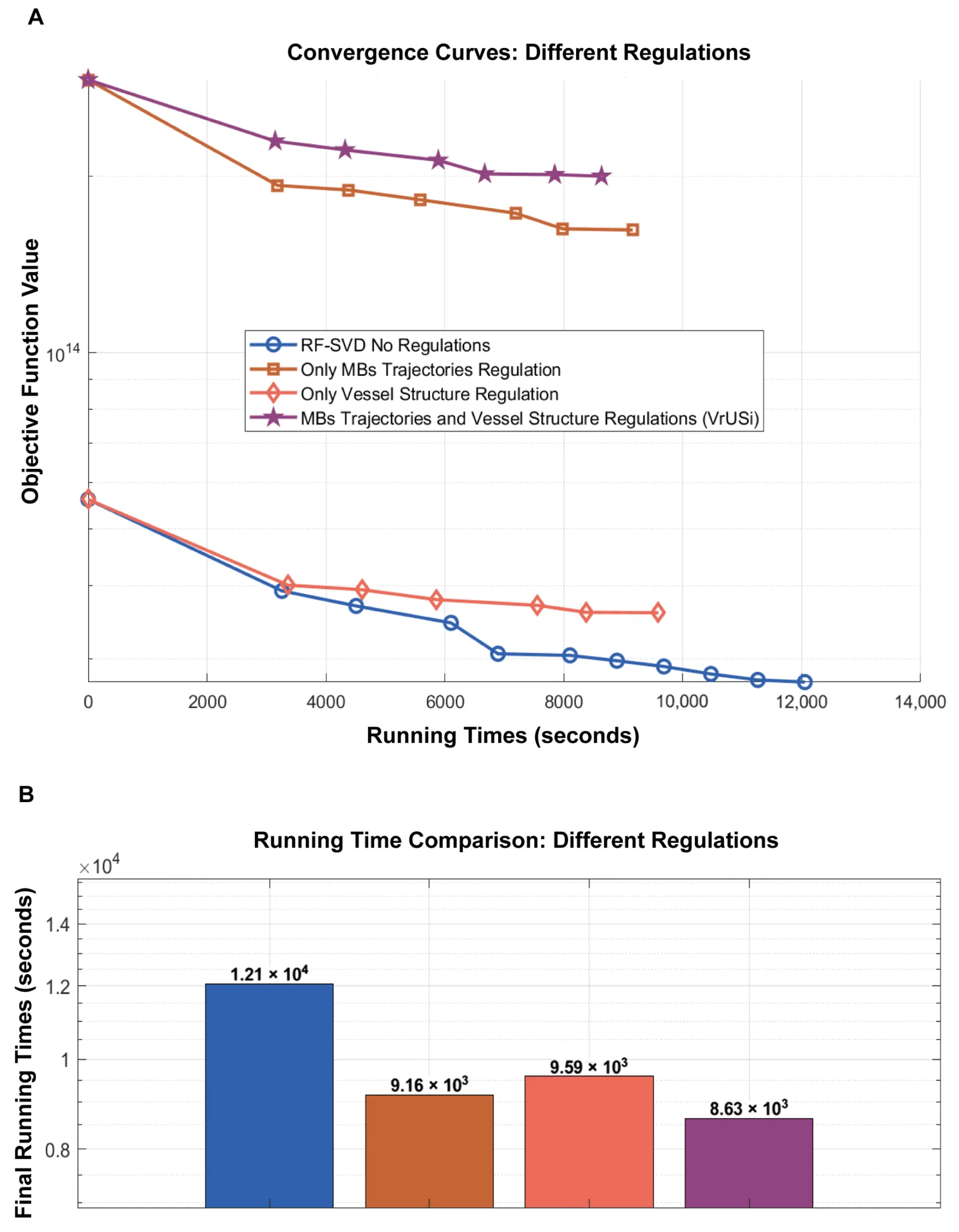
VrUSi running time under different regularization settings. (**A**) The running time and convergence of the objective function of VrUSi under different regularization settings. (**B**) The running time of VrUSi under different regularization settings.

Figure 9 further compares the convergence behavior and computation time of the optimization under different conditions. Figure 9A,B show that when raw RF data serve as the input, the convergence time is longer. When a single spatiotemporal constraint is imposed, the convergence time is effectively shortened. When both constraints are used together, the convergence time is further reduced.

### 4.6. Parameter Sensitivity Analysis of Compounding Angles

We designed an experiment to characterize the sensitivity of VrUSi to a reduced number of compounding angles within the available dataset. This experiment focused on a scenario with reduced transmissions (i.e., a lower number of plane-wave compound angles), which implies a lower CNR and higher noise.

We systematically reduced the number of plane-wave compound angles (from 5 angles down to single-angle transmission) and compared the reconstruction performance of VrUSi versus cUSi under identical conditions to evaluate our method with limited data and low CNR.

**Figure 10:**
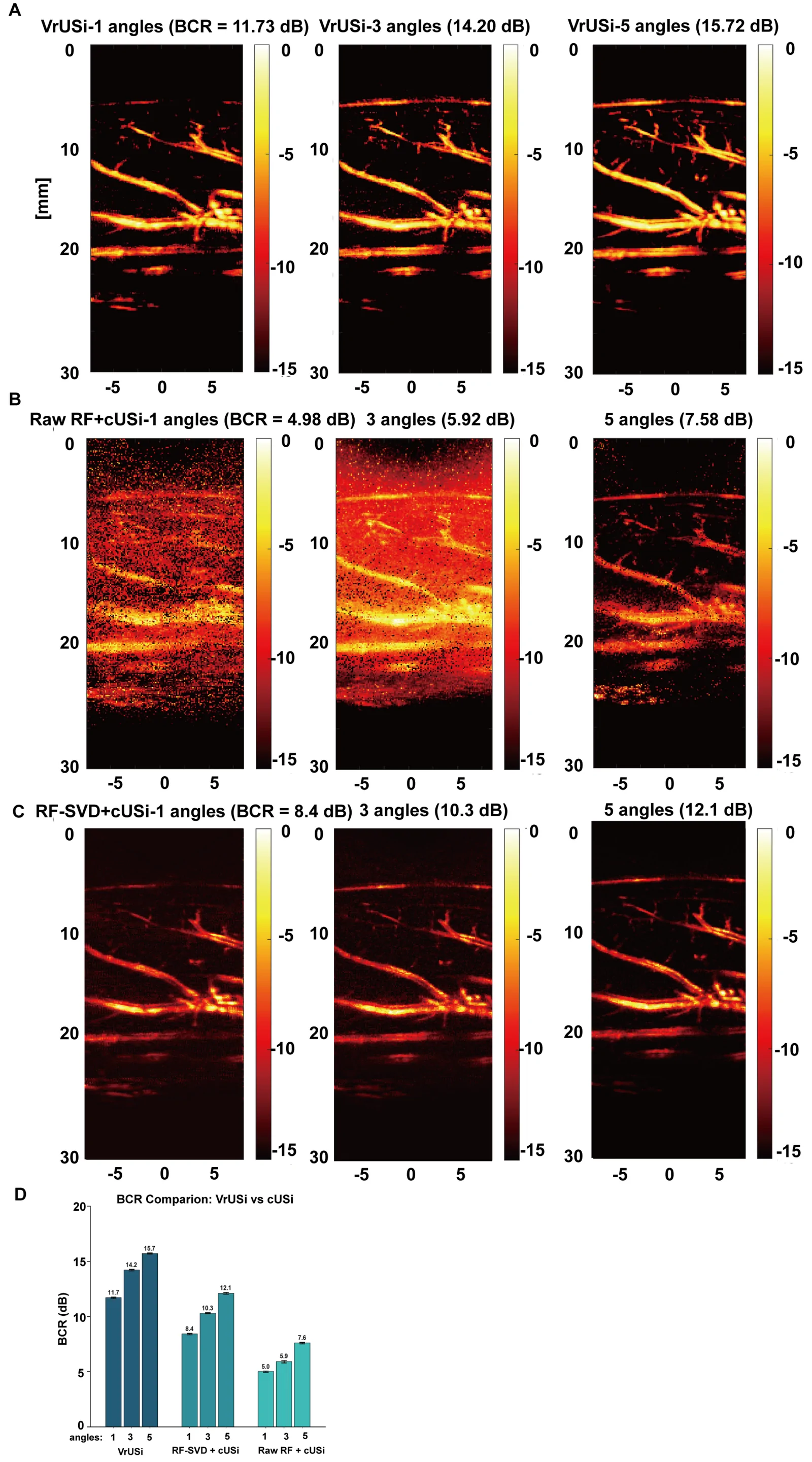
VrUSi and cUSi reconstruction results under different angles. (**A**) VrUSi bloodflow imaging results under 1, 3, and 5 angle plane wave compounding conditions. (**B**) cUSi (Raw RF) blood flow imaging results under 1, 3, and 5 angle plane wave compounding conditions. (**C**) cUSi (RF SVD) blood flow imaging results under 1, 3, and 5 angle plane wave compounding conditions. (**D**) Comparison of BCR indicators of VrUSi and cUSi under different angles.

Results are shown in Figure 10. Figure 10A shows that in the single-angle subset, VrUSi yielded a higher image-domain BCR than cUSi. Compared with the five-angle result, fewer fine vascular structures were visible and vascular continuity was reduced.

Figure 10B shows that, when only a single angle was used for cUSi (Raw RF data), the BCR decreased, background noise increased, and fewer continuous vascular structures were visible. As the number of angles increased, the BCR and the visible continuity of vessel-associated signals increased within this dataset.

Figure 10C shows cUSi reconstruction using data after RF-SVD. As the number of angles increased, background noise was more effectively suppressed, resulting in a higher BCR. Compared with cUSi reconstruction using raw RF data, RF-SVD produced lower image-domain background levels and a higher BCR under the tested settings.

Figure 10D shows that the BCR for both VrUSi and cUSi increases with more angles and that VrUSi yielded a higher BCR than cUSi at each tested angle count in this dataset. This result is an angle-count sensitivity observation and is not evidence of robustness across independent acquisitions.

### 4.7. Parameter Sensitivity Analysis of Downsampled Frame Rates

In this study, the data were acquired at 500 Hz, and optimization was performed on 1000-frame blocks. The same acquisition was retrospectively temporally downsampled to effective sampling rates of 250, 100, and 50 Hz. These datasets were not obtained from independent acquisitions performed at lower frame rates.

**Figure 11:**
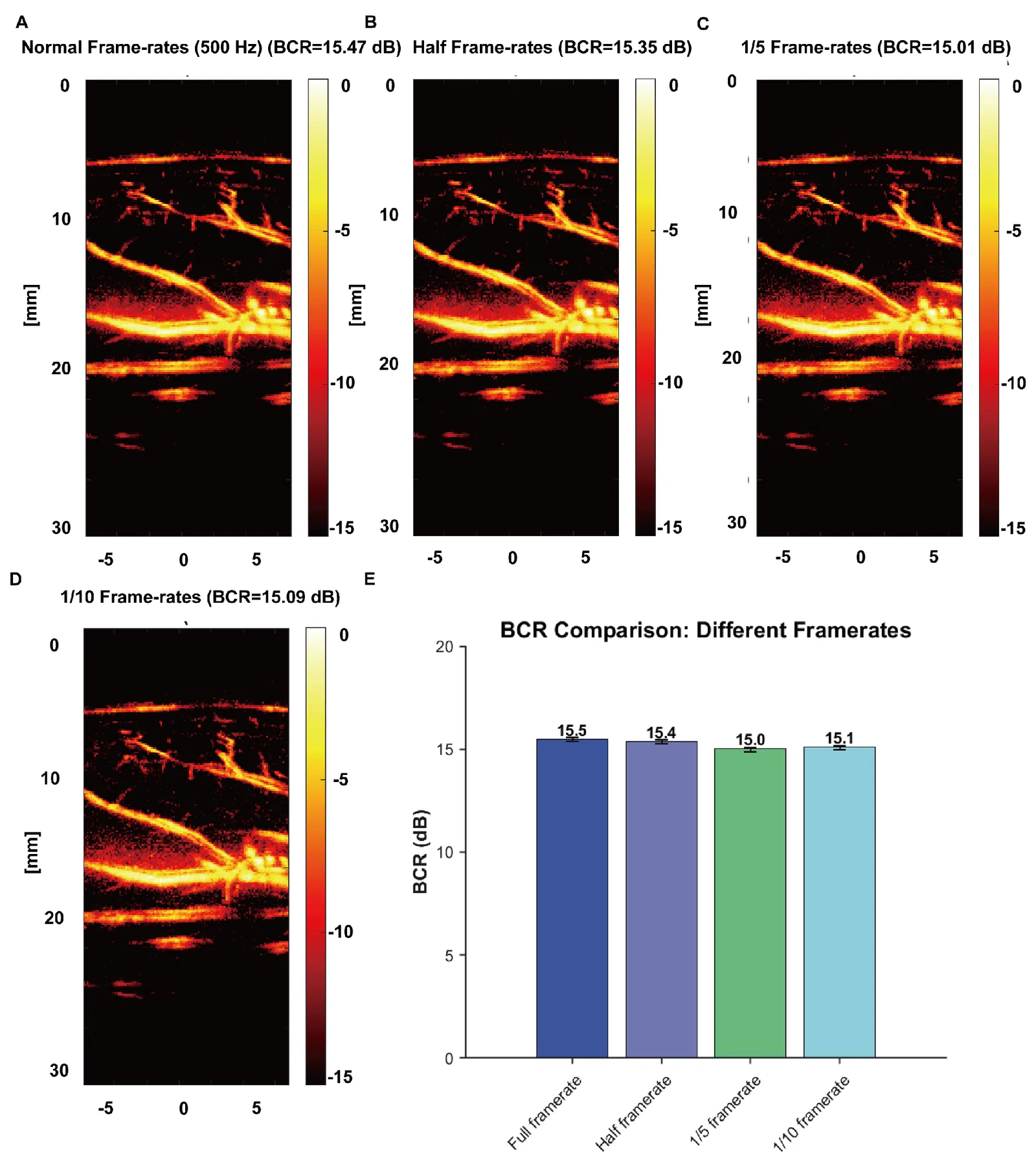
VrUSi imaging results at different downsampled frame rates. (**A**) Original frame rate (500 Hz). (**B**) One half of the original frame rate (250 Hz). (**C**) One fifth of the original frame rate (100 Hz). (**D**) One tenth of the original frame rate (50 Hz). (**E**) Comparison of BCR values at different downsampled frame rates. All reduced-rate datasets were generated by retrospective temporal downsampling of the same 500-Hz acquisition.

Figure 11A–D show the lower-limb reconstruction results from the original 500-Hz acquisition and from its one-half, one-fifth, and one-tenth temporally downsampled subsets, respectively. Figure 11E quantitatively compares the BCR values of the resulting flow-related images. The BCR values changed little across these retrospectively downsampled subsets within this single acquisition. This observation does not establish performance or robustness for data acquired prospectively at lower frame rates.

However, it should be noted that the downsampling strategy cannot fully represent reconstruction from data acquired at different frame rates. In our downsampling experiments, the acquisition duration was fixed at 2 s, corresponding to 1000 frames at the original frame rate. After downsampling, the number of reconstructed frames decreased. For example, 1/10 downsampling resulted in only 100 reconstructed frames. This reduction in frame number may affect the reconstruction results. Therefore, these results characterize sensitivity only to retrospective temporal downsampling of one acquisition; validation requires independent data acquired prospectively at predefined frame rates.

### 4.8. Parameter Sensitivity Analysis of Data Collection Duration

In contrast-enhanced ultrasound imaging, the MB concentration may change over time after contrast agent injection. In the present experiment, however, MB concentration was neither measured nor independently controlled. We therefore compared the first and last 1000-frame windows only to examine sensitivity to post-injection timing within the same lower-limb acquisition (frame rate = 500 Hz, total frames = 5000).

**Figure 12:**
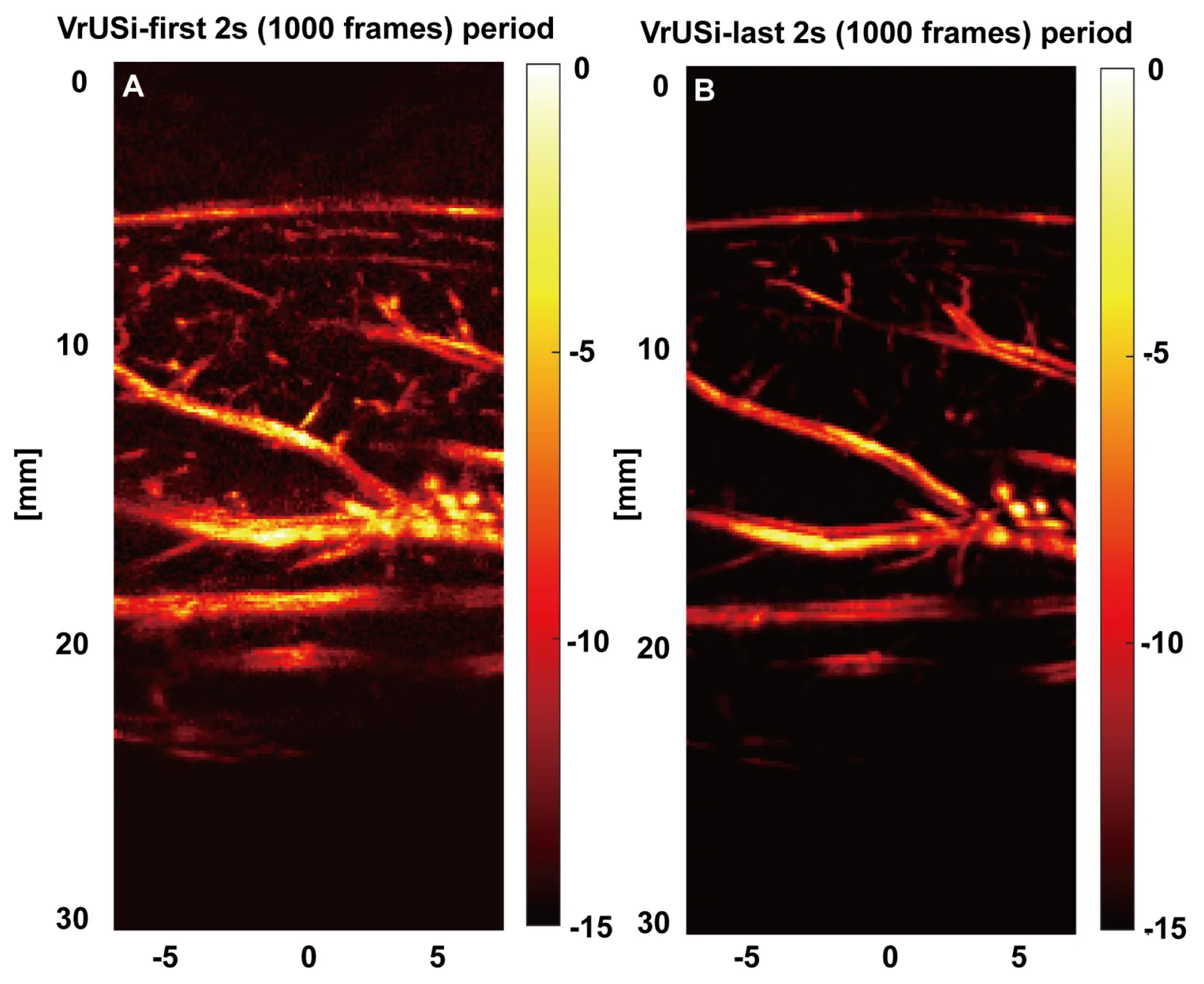
VrUSi reconstruction results under different periods. (**A**) VrUSi reconstruction results of the first 2s (1000 frames) period. (**B**) VrUSi reconstruction results of the last 2s (1000 frames) period.

Figure 12 shows that flow-related structures remained visible in both analyzed temporal windows. During the first 2-s period, the image contained a higher background level and more visible small vessel-associated structures. During the last 2-s period, the measured signal-to-noise ratio was higher, while the main vascular structures remained visible. These descriptive differences do not constitute a controlled microbubble-concentration experiment or demonstrate robustness to different concentrations. Supplementary Video S1 presents the reconstruction results under first and last 2-s periods.

However, it should be noted that only 10 s of data was acquired at a frame rate of 500 Hz. The data were collected after a single MB injection, without repeated acquisitions at different MB concentrations. Therefore, VrUSi was evaluated only for reconstruction over the full acquisition period following a single-dose injection. Its imaging performance at different MB concentrations requires further validation.

### 4.9. Running Time and Device Information

For the single-frame computational method cUSi, it is necessary to independently and sequentially complete T image reconstructions (solving the inverse problem) to obtain T frames of B-mode images, which are then processed through SVD filtering and other steps to finally synthesize a single flow image. Assuming each image has N pixels and requires $k_{single}$ iterations, the computational complexity of completing T frames of image reconstruction for the cUSi algorithm is: $Complexity\left(cUSi\right)=T\times \left[k_{single}\times O\left(N\right)\right]=O\left(T*k_{single}*N\right)$.

VrUSi solves an optimization problem with N∗T variables. Under the implementation and parameter settings evaluated here, the spatiotemporal regularization terms were associated with fewer iterations than framewise reconstruction. The numerical condition number was not evaluated, so no general claim of improved conditioning is made. The required number of iterations may therefore be reduced in this experiment ($k_{multi}<k_{single}$). The computational complexity of VrUSi is: $Complexity\left(VrUSi\right)=O\left(T*k_{multi}*N\right)$.

**Figure 13:**
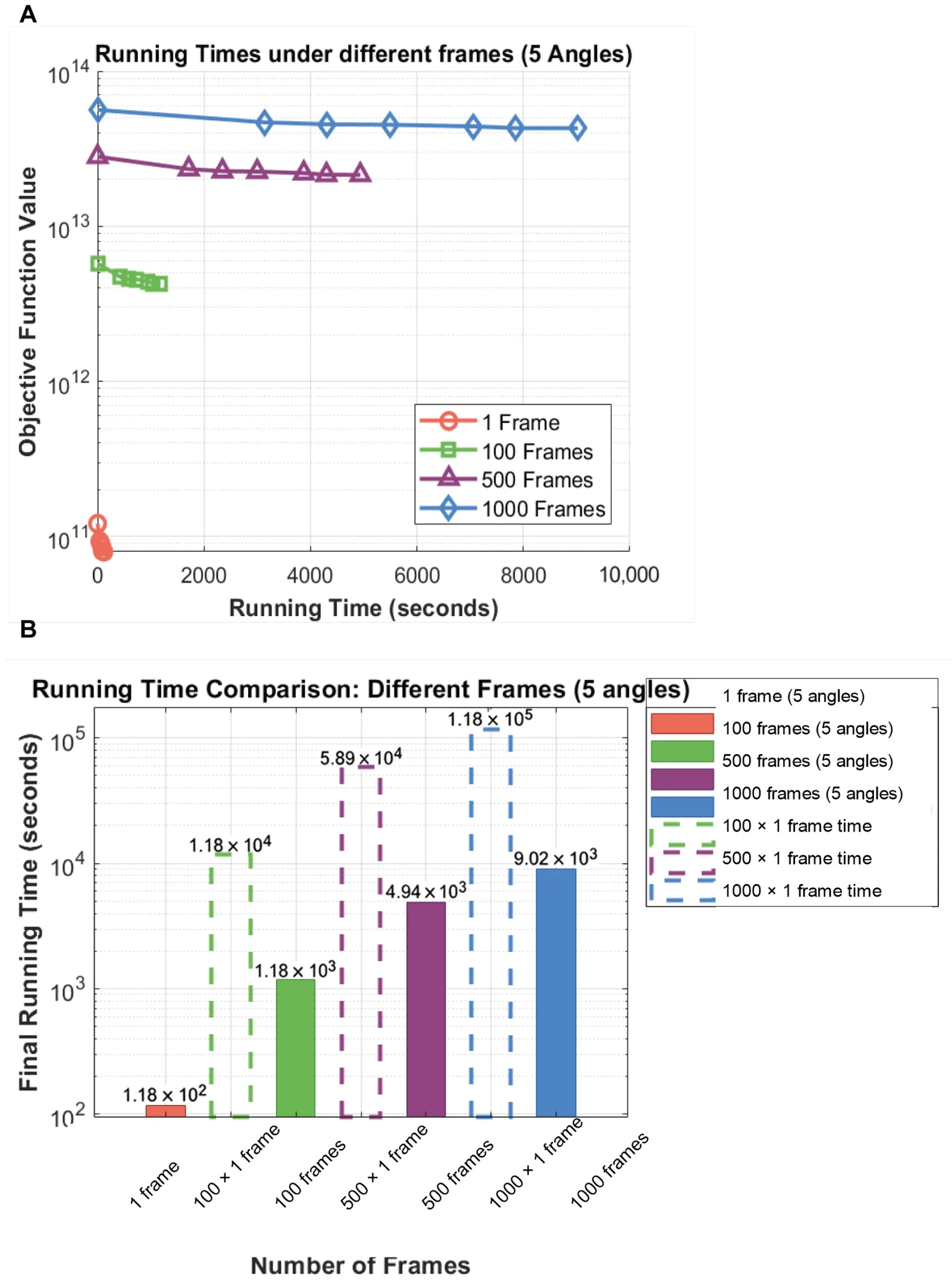
Comparison of the running times of VrUSi and cUSi. (**A**) The running time and convergence of the objective function of VrUSi under different frame numbers conditions. (**B**) The running time of VrUSi under different frame numbers compared with that of cUSi.

Figure 13A,B report the objective value against elapsed time for different frame counts. On the reported hardware, one cUSi frame required approximately 118 s, so sequential reconstruction of 1000 frames was estimated to require 118,000 s. The measured 1000-frame VrUSi run required approximately 8630 s, corresponding to a 13.7-fold reduction relative to that extrapolated framewise time. This is a measured runtime comparison, not a change in asymptotic complexity. Moreover, 8630 s (approximately 2.4 h) was required to reconstruct 2 s of acquired data, so the present implementation is offline and not real time.

**Figure 14:**
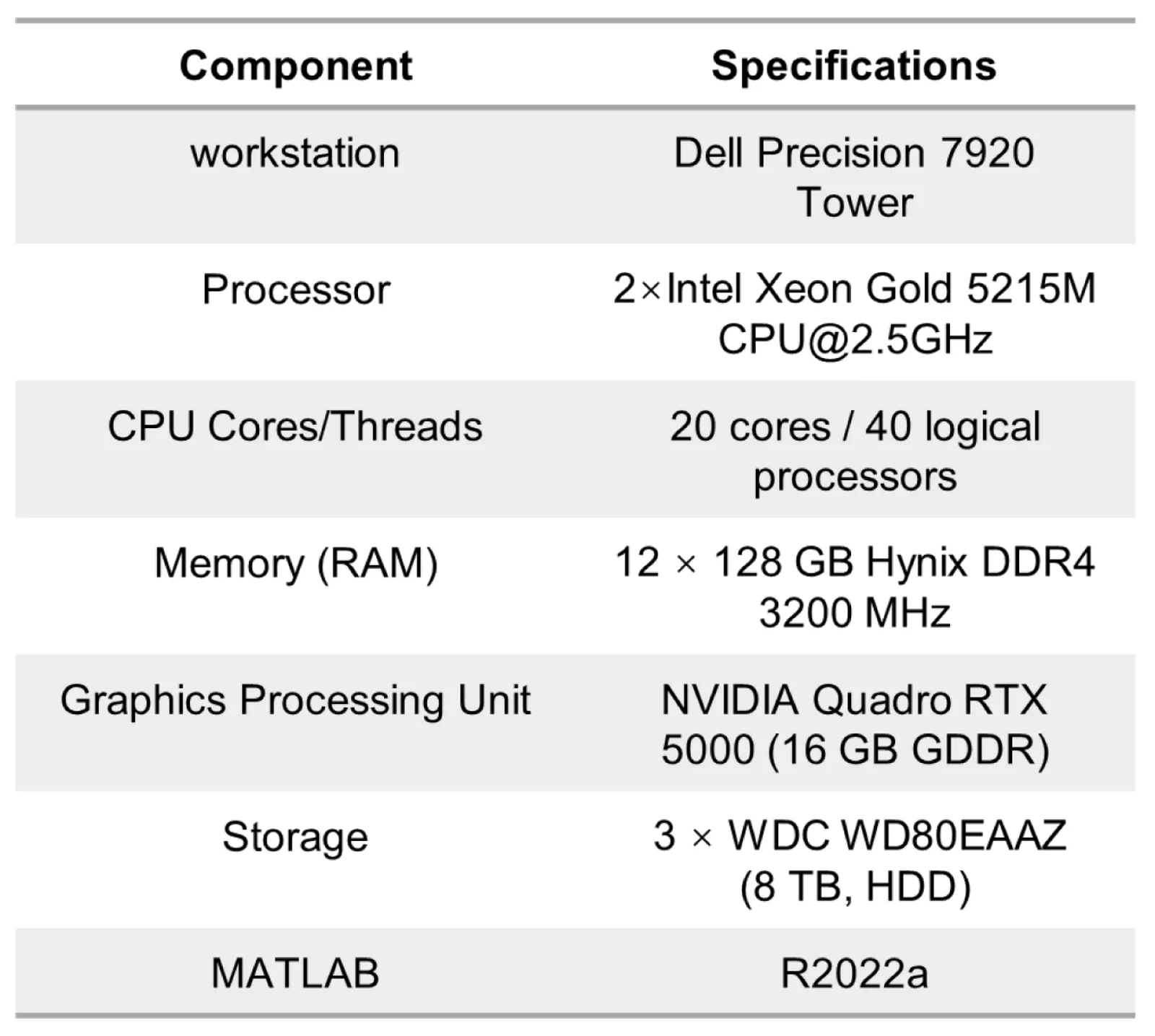
The device information for implementing and running VrUSi.

The device information used to run and implement VrUSi in this study is provided in Figure 14.

## 5. Discussion

Researchers have developed cUSi for microflow imaging [13,14,15,21], yet multi-frame sparsity information, widely used to improve MB localization and tracking in ULM, remains underexploited in cUSi [17,18,19,20]. In conventional PWI, tissue signals in raw RF data severely reduce the sparsity of data, preventing direct imposition of multiple frames sparsity information as regularization terms [32,33,34].

To address this, we apply SVD filtering to raw RF data. This attenuates tissue-dominated components, increases the relative contribution of retained flow-related components, and changes the observed convergence behavior. We further impose sparsity constraints on multiple frames, achieving multi-frame reconstruction of contrast-enhanced microflow ultrasound images.

It should be acknowledged that VrUSi imaging results are influenced by the regularization penalty parameter. When the penalty parameter is small, such as 0 or 0.002, VrUSi yields a slightly lower CNR than DAS, while the apparent axial FWHM must be interpreted separately because regularization can narrow the reconstructed profile. This is illustrated in Figure 4C,D and Figure 5C. At small penalty values, less low-amplitude background signal is suppressed; this explains the lower CNR but should not be used to interpret FWHM as a physical-resolution measurement.

L1 regularization suppresses low-intensity background signals and may narrow the reconstructed intensity distribution. Therefore, a higher BCR mainly indicates stronger background suppression under the specific acquisition conditions and parameter settings. We acknowledge that the BCR may become very large when the background signal approaches the numerical or detection floor. Such values should likewise be interpreted as background suppression approaching the detection limit. They do not indicate an unlimited increase in physical contrast. Therefore, the reduced FWHM should also be interpreted as an improvement in apparent profile compactness rather than a direct equivalent improvement in the intrinsic physical resolution of the imaging system.

Motion sensitivity is an important limitation of the current VrUSi framework. The in vivo experiment was performed in the rabbit lower limb under relatively low-motion conditions. Therefore, the present results support feasibility only in the tested rabbit lower-limb setting. They do not establish equivalent performance in organs affected by respiration, cardiac pulsation, or complex tissue deformation, such as the liver, kidney, or heart. Performance in other anatomical sites and motion conditions remains unknown and requires direct evaluation.

Our method is also affected by tissue motion because SVD clutter filtering is motion-sensitive and may introduce artifacts, impacting sparsity constraints [27,34,35]. No motion-correction experiment was performed, and VrUSi should not be considered motion-robust. No conclusions can be drawn from the present data regarding performance in the brain or in organs affected by respiration, cardiac pulsation, or complex deformation. Extension to these settings will require motion estimation, motion-compensated reconstruction at the RF-data level, and direct experimental validation.

We evaluated the sensitivity of the regularization parameter τ. As shown by muscle flow phantom results, the image-domain signal distribution depends on τ. A large τ (e.g., 0.2) causes excessive signal suppression. Conventional cUSi preserves high-intensity tissue background [13,14], so increasing τ severely suppresses flow-related signals. In contrast, VrUSi pre-filters tissue signals before reconstruction, allowing flow-related signal retention at the tested values of τ. In the single in vivo lower-limb acquisition, the BCR and apparent-profile FWHM were reported for τ values from 0 to 0.02. Their limited variation within this dataset is not evidence of robustness across independent acquisitions or parameter settings. Figure 7 presents specific in vivo imaging results. As the regularization penalty parameter increased, cUSi produced lower flow-related signal intensity and higher relative background levels. Theoretically, a larger penalty parameter should suppress more weak signals, i.e., background noise. A major factor behind this phenomenon is that conventional cUSi first computes a B-mode image and then applies SVD filtering. During the cUSi reconstruction of the B-mode image, a larger penalty parameter suppresses the relatively weaker blood flow signals. The subsequent SVD filtering attenuates high-intensity static tissue signals while retaining moving blood flow signals and flickering background noise. Consequently, a larger penalty parameter suppresses blood flow signals more severely, making it harder to distinguish blood flow from background noise after SVD filtering. Thus, under the tested settings, increasing the cUSi penalty parameter decreased the BCR and altered the image-domain balance between flow-related signal and background.

The selection of the regularization parameter τ is another limitation of VrUSi. τ was selected empirically based on the reconstruction results. An independent and objective parameter-selection criterion was not used. We therefore acknowledge that the selected τ may not be directly applicable to different imaging targets, acquisition conditions, or system configurations. Parameter selection may be affected by the relative intensities of tissue and blood-flow signals, noise level, MB concentration, number of frames, and model errors in the system matrix. This empirical selection limits the reproducibility and transferability of VrUSi across new datasets. Future studies will investigate more objective parameter-selection strategies. For example, the L-curve method could be used to balance the data-fidelity and regularization terms. Alternatively, the discrepancy principle could be applied according to the estimated noise level.

We constructed a pulse—echo matrix for reconstruction and measured the EIR using a hydrophone [15]. Due to equipment constraints, a short excitation pulse (duty cycle 0.3, 0.5 cycles, 7.8 MHz) provided insufficient energy, yielding noise-corrupted EIR measurements. To overcome this, we excited multiple elements to focus at a 10 mm depth and placed the hydrophone at the focal point to record the EIR. It should also be noted that the EIR measured in this work includes the EIR of the hydrophone itself. The hydrophone was calibrated at all frequency components, so the measured values closely match the true values. Therefore, the influence of the hydrophone’s own EIR on the measurement results is not further considered in this study.

However, the EIR measurement procedure is a limitation of the present study. In the experiment, the short-pulse response from a single element had a low signal-to-noise ratio. As a practical compromise, multiple elements were focused during transmission, and the response was recorded using a hydrophone positioned at the focal point. Therefore, the measured waveform contains not only the responses of the transducer elements, excitation electronics, and receive chain but also the effects of multi-element focusing and the hydrophone transfer response. The EIR in our study should be regarded as an effective composite response of the experimental system under the specific measurement conditions, rather than as an exact single-element EIR. We acknowledge that constructing the system matrix *P* using this composite response may introduce systematic model errors. These errors propagate into the forward model and may cause discrepancies between the predicted RF signals and the actual signals received by a single element.

The forward model in our study assumes a linear relationship between the received signals and the scatterer distribution to be reconstructed. Therefore, it cannot describe nonlinear MB oscillations, harmonic generation, or other nonlinear scattering behaviors. Although contrast-enhanced data were used in this study, contrast-enhanced describes only the experimental acquisition condition. It does not imply that the VrUSi forward model is intrinsically specific to MBs. In the current framework, RF-SVD separates tissue and flow-related scattering signals mainly according to differences. It does not exploit the nonlinear acoustic responses specific to MBs. Therefore, the signals retained and reconstructed by VrUSi are more accurately described as flow-related signals. We acknowledge that the method cannot strictly distinguish MB echoes from red-blood-cell scattering or signals produced by other moving scatterers. Tissue or probe motion may also occupy overlapping signal subspaces and affect the separation of flow-related components.

In addition, we did not evaluate blood-flow imaging without contrast agents, where the signal originates mainly from red-blood-cell scattering. Therefore, the sensitivity, clutter-suppression performance, and reconstruction behavior of VrUSi in contrast-free microflow imaging remain to be established. In principle, RF-SVD may also separate signals from moving red blood cells. However, flow echoes are generally weaker under contrast-free conditions. They may also be more sensitive to noise, tissue motion, and parameter selection. Future studies should directly compare contrast-enhanced and contrast-free data acquired from the same imaging region using matched acquisition settings. Future work may combine MB-dynamics models with nonlinear acoustic-propagation models to construct a system matrix that represents nonlinear MB responses.

In our study, a dense imaging grid of 285 axial × 128 lateral pixels with half-wavelength spacing (0.1 mm) covered 12.8 mm × 28.5 mm. While resolution beyond half-wavelength is termed super-resolution, we conservatively keep half-wavelength spacing, as we do not aim to investigate super-resolution here [17,36,37,38]. Our experiments evaluated VrUSi only with multi-angle PWI. No conclusion can be drawn about its performance with random, structured, or other complex sound fields without direct testing.

The parameter sensitivity analysis was based on two types of data. First, different effective temporal-sampling datasets were generated by temporally downsampling the same original acquisition. Second, different post-injection time windows were selected from a single acquisition in one animal. These analyses were used to preliminarily examine the sensitivity of VrUSi to temporal sampling and post-injection timing. Within this single acquisition, the reported image-domain measurements varied by a limited amount across the selected post-injection windows and retrospectively downsampled subsets. However, these analyses are not equivalent to independent acquisitions performed at different frame rates. They also do not constitute a controlled MB-concentration experiment. Temporal downsampling changes both the effective number of frames and the temporal sampling density. Differences between post-injection windows may also reflect multiple factors, including MB concentration, hemodynamic variation, and signal decay. We therefore acknowledge that the performance of VrUSi under different actual acquisition frame rates and controlled MB concentrations requires further investigation. Future studies should use independent acquisitions with predefined frame rates and contrast-agent doses.

Typical single-frame methods (e.g., SMIF [15], cUSi [13,14]) solve for a B-mode image with an L1 constraint. In contrast, VrUSi operates on a spatiotemporal matrix of multiple frames and imposes sparsity on multiple frames. Bar-Zion et al. [21] use PI in contrast mode to obtain tissue-free blood flow signals and constrain temporal variance, which corresponds to vascular structures. We acquire raw data containing tissue in PWI mode and apply SVD filtering for clutter suppression, making VrUSi compatible with the high-frame-rate PWI data evaluated here. Moreover, we construct the system matrix using a hydrophone-measured probe pulse response. This waveform is treated as an effective composite system response under the specific measurement conditions; because model-mismatch sensitivity was not quantified, no claim of enhanced practical reliability is made.

Many cUSi methods exploit sparsity in frequency or spatial domains. Ozkan et al. reviewed various regularization terms for ultrasound computational imaging [11]. Goudarzi et al. and Yan et al. introduced denoising algorithms into regularization terms [16,20]. In ULM, spatiotemporal priors have been used to improve MB localization and tracking. Leconte et al. tracked trajectories before localization [19], Yin and Zhang et al. proposed TPSF to optimize imaging based on MB trajectory features [17,18], and Yan et al. extended deconvolution to 3D spatiotemporal data with trajectory and denoising constraints [20]. Inspired by these works, we introduce spatiotemporal priors into the beamforming inverse problem. However, the pixelwise temporal standard deviation is equivalent to a group norm of mean-centered samples and is invariant to any permutation of the frames. It can encourage a compact distribution of temporal fluctuations, but it cannot distinguish a smooth trajectory from a randomly ordered sequence. Explicit temporal evolution would require an order-sensitive term, such as temporal total variation, an optical-flow or motion-guided constraint, or a state-space trajectory model. Evaluating such alternatives is an important direction for future work.

Recent studies on computational ultrasound microflow imaging have followed four main directions. These include physics-based reconstruction, clutter separation, learning-based signal enhancement, and MB localization in ULM. Brown et al. achieved four-dimensional computational imaging of mouse brain hemodynamics. They used complex encoded acoustic fields and a calibrated forward model. Their study showed that model-based reconstruction can reduce hardware sampling requirements at the cost of greater computational complexity [13]. Chen et al. extended low-rank tissue and sparse-flow decomposition to an online IRLS-rPCA framework. Their method enables rapid clutter suppression in consecutive beamformed frames [39]. Lan et al. proposed deep power-aware tunable weighting. It enhances ultrafast power Doppler and ULM images after DAS beamforming [40]. Huang et al. constructed self-supervised training pairs from complementary steering-angle subsets. Their method suppresses noise in beamformed RF signals [41]. In ULM, Shin et al. used context-aware learning to improve microbubble localization at high concentrations [42]. Yan et al. introduced multi-frame deconvolution with temporal total variation. Their method improves localization accuracy and temporal consistency [20].

VrUSi differs from these methods in several respects. It does not require training data. It also does not localize or track individual MBs. Instead, VrUSi applies SVD clutter filtering to raw channel RF data. It then jointly reconstructs multiple frames using a physics-based pulse—echo model. Its main distinction is that clutter separation is performed before model-based beamforming. This increases the relative contribution of weak flow signals in the sparse inverse problem. However, the current pixelwise temporal-standard-deviation regularizer is invariant to frame permutation. Unlike the temporal regularization used by Yan et al., it does not encode temporal order or motion trajectories [20]. VrUSi also lacks explicit motion compensation. In contrast, Ouzir et al. jointly estimate blood flow, tissue components, and tissue motion [43]. Moreover, VrUSi does not yet meet the computational requirements of online imaging. This is an important limitation compared with the methods of Chen, Lan, and Huang et al. [39,40,41]. We therefore regard VrUSi as an offline feasibility study of raw-RF-domain preprocessing and multi-frame computational microflow reconstruction.

The clinical workflow is currently constrained by both data access and computation. Many clinical scanners export beamformed IQ data or images rather than the raw channel RF data required here. On the reported hardware, reconstructing 2 s of 500-Hz data required 8630 s. This represents a 13.7-fold reduction in runtime relative to the extrapolated sequential cUSi time, but it is not real time. At present, VrUSi cannot yet achieve real-time reconstruction or be directly integrated into clinical ultrasound systems.

Ultrasound blood flow imaging has become a new tool for many fields [44,45]. Here we applied SVD filtering to raw RF data and then imposed constraints on multiple frames to achieve blood flow imaging. Our VrUSi method offers a technical reference for researchers requiring complex beamforming in ultrasound blood flow imaging.

## Figures and Tables

**Table 1:** Summary of quantitative results across the simulation, sparse MB, flow phantom, and in vivo experiments. Values are reported as the mean value and standard deviation. For simulation FWHM, the statistics were calculated across scatterers at multiple depths. For sparse MB FWHM, they were calculated across different temporal window reconstructions from the same acquisition. For the flow phantom and in vivo experiments, they were calculated across temporal windows within the respective single acquisitions. Bold text indicates method names, column headings, and metric labels and does not denote statistical significance or superior performance.

Method	Simulation	Sparse MB Imaging	Flow Phantom	In Vivo
**DAS**	**CNR (dB)** Region 1: 6.30±0.10 Region 2: 7.60±0.10 **FWHM (mm)** Axial: 0.28±0.05 Lateral: 0.46±0.05	**FWHM (mm)** 0.34±0.10	–	**FWHM (mm)** 0.56±0.10 **BCR (dB)** 18.92±0.10
**Raw RF + cUSi**	–	–	**BCR (dB)** −1.21±0.05 (τ=0.02)	**FWHM (mm)** 0.47±0.10 **BCR (dB)** 13.52±0.10 **Computation time (s)** $1.18\times 10^{5}$
**RF SVD + cUSi**	–	–	–	**FWHM (mm)** 0.45±0.10 **BCR (dB)** 20.84±0.10 **Computation time (s)** $1.13\times 10^{5}$
**VrUSi**	**CNR (dB)** Region 1: 7.69±0.10 Region 2: 9.63±0.10 **FWHM (mm)** Axial: 0.18±0.05 Lateral: 0.21±0.05	**FWHM (mm)** 0.24±0.05	**BCR (dB)** 21.20±0.05 (τ=0.02)	**FWHM (mm)** 0.42±0.10 **BCR (dB)** 22.75±0.10 **Computation time (s)** $8.63\times 10^{3}$

Dashes indicate that the corresponding metric was not calculated for that method. The simulation and sparse-MB imaging experiments were designed to compare the image-domain measurements obtained with computational reconstruction and DAS. Therefore, only DAS and the proposed method were compared. The phantom experiment was designed to investigate the effects of tissue signals on cUSi and the proposed method. FWHM denotes full width at half maximum, CNR denotes contrast-to-noise ratio, and BCR denotes blood-to-clutter ratio.

## Data Availability

The original contributions presented in this study are included in the article. Further inquiries can be directed to the corresponding author. Raw RF data, system matrix P data, and EIR matrix data are available upon request. Please contact the corresponding author. The public code repository for VrUSi is: https://github.com/lddmonster/Codes_for_VrUSi.git (accessed on 22 September 2026).

## Supplementary Materials

The following supporting information can be downloaded at https://www.mdpi.com/article/10.3390/jimaging12100511/s1, File S1: The ARRIVE guidelines 2.0: author checklist; Video S1: VrUSi reconstruction results under first and last 2-s periods.
